# Defense arsenal of the strict anaerobe *Clostridioides difficile* against reactive oxygen species encountered during its infection cycle

**DOI:** 10.1128/mbio.03753-24

**Published:** 2025-03-20

**Authors:** Aurélie Lotoux, Léo Caulat, Catarina Martins Alves, Carolina Alves Feliciano, Claire Morvan, Filipe Folgosa, Isabelle Martin-Verstraete

**Affiliations:** 1 Institut Pasteur, Université Paris Cité, UMR CNRS 6047, Laboratoire Pathogenèse des Bactéries Anaérobies 555089 , Paris, Île-de-France, France; 2 Instituto de Tecnologia Química e Biológica António Xavier, Universidade Nova de Lisboa 98819 , Oeiras, Lisbon, Portugal; 3 Institut Universitaire de France 89211 , Paris, Île-de-France, France; University of Delaware, Newark, Delaware, USA

**Keywords:** peroxidase, superoxide reductase, rubrerythrin, stress response, PerR, Sigma B, Spx

## Abstract

*Clostridioides difficile*, a strict anaerobe, is the major cause of antibiotic-associated diarrhea. This enteropathogen must adapt to oxidative stress mediated by reactive oxygen species (ROS), notably those released by the neutrophils and macrophages recruited to the site of infection or those endogenously produced upon high oxygen (O_2_) exposure. *C. difficile* uses a superoxide reductase, Sor, and several peroxidases to detoxify ROS. We showed that Sor has a superoxide reductase activity *in vitro* and protects the bacterium from exposure to menadione, a superoxide donor. After confirming the peroxidase activity of the rubrerythrin, Rbr, we showed that this enzyme together with the peroxiredoxin, Bcp, plays a central role in the detoxification of H_2_O_2_ and promotes the survival of *C. difficile* in the presence of not only H_2_O_2_ but also air or 4% O_2_. Under high O_2_ concentrations encountered in the gastrointestinal tract, the bacterium generated endogenous H_2_O_2_. The two O_2_ reductases, RevRbr2 and FdpF, have also a peroxidase activity and participate in H_2_O_2_ resistance. The *CD0828* gene, which also contributes to H_2_O_2_ protection, forms an operon with *rbr*, *sor*, and *perR* encoding a H_2_O_2_-sensing repressor. The expression of the genes encoding the ROS reductases and the CD0828 protein was induced upon exposure to either H_2_O_2_ or air. We showed that the induction of the *rbr* operon is mediated not only by PerR but also by OseR, a recently identified O_2_-responsive regulator of *C. difficile*, and indirectly by σ^B^, the sigma factor of the stress response, whereas the expression of *bcp* is only controlled by σ^B^.

**IMPORTANCE:**

ROS plays a fundamental role in intestinal homeostasis, limiting the proliferation of pathogenic bacteria. *Clostridioides difficile* is an important enteropathogen that induces an intense immune response, characterized by the massive recruitment of immune cells responsible for secreting ROS, mainly H_2_O_2_ and superoxide. We showed in this work that ROS exposure leads to the production of an armada of enzymes involved in ROS detoxification. This includes a superoxide reductase and four peroxidases, Rbr, Bcp, revRbr2, and FdpF. These enzymes likely contribute to the survival of vegetative cells of *C. difficile* in the colon during the host immune response. Distinct regulations are also observed for the genes encoding the ROS detoxification enzymes allowing a fine tuning of the adaptive response to ROS exposure. Understanding the mechanisms of ROS protection during infection could shed light on how *C. difficile* survives under conditions of an exacerbated inflammatory response.

## INTRODUCTION

Oxygen (O_2_)-derived compounds, such as reactive oxygen species (ROS), are important signaling molecules, but they can also cause irreversible damages to proteins at high concentrations (1–3). ROS include various compounds, such as the radical superoxide (O_2_^•−^) and the non-radical hydrogen peroxide (H_2_O_2_), which are formed from the partial reduction of O_2_. These molecules are the precursors of even more reactive compounds such as hydroxyl radical or hypochlorous acid (HOCl). In the presence of O_2_, ROS can be produced endogenously and intracellularly by eukaryotic and prokaryotic cells (4, 5). In the gut, ROS can result from the metabolic activity of the microbiota, the metabolism of eukaryotic cells and host-mediating immune response (6). During bacterial infections such as those due to *Clostridioides difficile*, the recruitment of circulating innate and adaptive immune cells in the colon, mainly neutrophil and macrophage cells, leads to the production of ROS, nitric oxide, reactive nitrogen species, and HOCl that contribute to the eradication of pathogens. The balance of the immune response is an important determinant of the severity of the infection (7).

*C. difficile* is a gram-positive, spore-forming obligate anaerobe, which is the leading cause of nosocomial diarrhea in adults in developed countries (8). *C. difficile* infection (CDI) often occurs following an antibiotic treatment that causes dysbiosis and a change in intestinal homeostasis (9, 10). Symptoms can range from severe diarrhea to pseudomembranous colitis or toxic megacolon, which can lead to the death of the patient (11, 12). During its infectious cycle, *C. difficile* is particularly exposed to oxidative stress. *C. difficile* spores face air and disinfectants, such as H_2_O_2_ and HOCl, which are used for their eradication. After the germination of spores in the gastrointestinal tract (GIT), vegetative cells produce two toxins, TcdA and TcdB, which are glycosyltransferases targeting small Rho GTPases. They alter the actin cytoskeleton of intestinal epithelial cells causing cellular detachment, enterocyte lysis, and thus the permeabilization of the gut epithelium. Toxins and tissue damage lead to the production of pro-inflammatory cytokines and chemokines triggering an intense inflammatory response with an important recruitment of neutrophils and macrophages that produce an oxidative burst (12). During dysbiosis, O_2_ tensions in the GIT increase as O_2_ is consumed less by epithelial cells (13). *C. difficile* is therefore exposed to O_2_ (14, 15). Along the GIT, a decreasing longitudinal gradient of O_2_ exists ranging from 4% to 5% in the small intestine, where spores germinate, to 0.1 to 0.4% in the lumen of the colon (16). A second lateral O_2_ gradient is present with an increase from the colonic lumen towards the mucus (1%–2%) and the tissues (5%) (17). To detoxify O_2_, *C. difficile* has four O_2_-reducing enzymes, two flavodiiron proteins (FdpA and FdpF), and two reverse-rubrerythrins (revRbr1 and revRbr2, which are primarily H_2_O_2_-reductases) (15, 18). O_2_ exposure can also trigger endogenous ROS production and oxidative stress (4, 5).

To colonize a host, pathogens need to detoxify exogenous or endogenous ROS. Several ROS detoxification enzymes exist in bacteria. O_2_^•−^ can be converted to H_2_O_2_ and O_2_ by O_2_^•−^ dismutases (SOD) or to H_2_O_2_ by O_2_^•−^ reductase (SOR) in the presence of NAD(P)H and a redox partner, usually a rubredoxin (Rd) and a NAD(P)H:Rd-oxidoreductase (NROR) (19, 20). H_2_O_2_ can then be converted into water and O_2_ by catalases or only into water by different classes of peroxidases. Rubrerythrins (Rbr) are peroxidases that reduce H_2_O_2_ using NAD(P)H as electron donor and redox partners similar to SORs (21). In addition, peroxiredoxins (Prx) are important scavengers of hydroxyperoxides in bacteria especially when catalases or glutathione-peroxidases are absent. Prxs notably include cysteine-dependent peroxidases such as the thiol-peroxidases and the bacterioferritin comigratory protein, Bcp (22–24). To function, Prx enzymes require reductants that are most frequently the thioredoxin (Trx) systems. In *C. difficile*, two enzymes are probably involved in the detoxification of O_2_^•−^, SodA and Sor (CD0827), and seven enzymes share similarities either with the peroxidases of *Clostridium acetobutylicum* (21) or with other H_2_O_2_ detoxifying enzymes. These enzymes correspond to two Rbrs (Rbr/CD0825 and Rbr2/CD2845), three catalases (CotCB, CotD, and CotG), and two Prxs (Bcp/CD1822 and CotE). Interestingly, Rbr2, CotE, SodA, and the three catalases are only produced during sporulation and are present in the spore, whereas the other enzymes likely function in vegetative cells. It is worth noting that three O_2_-reducing enzymes out of four, FdpF and the two revRbrs, have also peroxidase activity *in vitro* (18, 25). Recently, a mutant inactivated for *sor* in a strain of the ribotype 027 (RT027) has been shown to be more sensitive to air and to menadione, a O_2_^•−^ donor (26). Another study has also shown that the *rbr* and *sor* genes form an operon with *perR* and *CD0828*. This operon is controlled by the PerR repressor (27). PerR controls pathways involved not only in H_2_O_2_ detoxification in Bacillota but also O_2_ detoxification systems in *C. acetobutylicum* (28). In *Bacillus subtilis*, a Fe^2+^-dependent oxidation of a histidine of PerR in the presence of H_2_O_2_ inactivates this repressor, triggering the production of peroxidases and catalases (29, 30). In *C. acetobutylicum*, *perR* inactivation leads to prolonged aerotolerance and to a derepression of genes involved in oxidative stress response (28). A mutation in the *perR* gene is present in the *C. difficile* 630∆*erm* strain, which has an increased tolerance to O_2_. The T41A modification in the helix-turn-helix motif of PerR (indicated as *perR*_mut_) modifies its binding to the promoter region of the *rbr* operon and leads to its overexpression (27). In addition to PerR, other factors, such as σ^B^ and OseR (15), could control the expression of genes involved in oxidative stress response. Indeed, a *sigB* mutant of *C. difficile* is more sensitive to exposure to low O_2_ tension or to H_2_O_2_ (31), whereas OseR is a newly described O_2_-sensing regulator of *C. difficile* that represses the expression of O_2_-reductase genes upon long-term exposure to 1% O_2_ (15).

In this work, we studied the oxidative stress defense of *C. difficile*. First, the physiological role of the main enzymes involved in ROS detoxification (Rbr and Sor) and their enzymatic activities have been studied. We also evaluated the ability of CD0828 encoded by the *rbr* operon to act as an electron donor for Rbr and Sor. Second, we tested the role of the Prx, Bcp, in the response to H_2_O_2_. Finally, we compared the regulation of the expression of the *bcp* gene and the *rbr* operon.

## RESULTS

### The *rbr* operon of *C. difficile*

The *rbr* operon encodes proteins with a role in ROS detoxification and the H_2_O_2_-sensing repressor, PerR, which likely controls the expression of this operon (27, 32). Rbr belongs to a class of enzymes with a peroxidase activity widespread in anaerobes and Clostridia. Rbr has a four-helix bundle structure, harboring a diiron catalytic center in its N-terminal part and a short-chain Rd-like domain marked by two C(xx)C motifs separated by 12 residues, responsible for the iron binding, in its C-terminal part (Fig. S1A) (33). The desulfoferrodoxin (its initial name), CD0827/Sor*,* is a 2Fe-SOR (19, 20, 34). The 2Fe-SORs contain two iron centers, a desulforedoxin-like center (center I), which is composed of an iron coordinated by four cysteines with a C(xx)C(x)_15_CC binding motif and a catalytic neelaredoxin-like center (center II), consisting of an iron coordinated by four equatorial histidines and an axial cysteine, in the reduced state, with a binding motif (E)(K)H(x)_19-27_H(x)_5_H(x)_40-63_C(x)_2_H (Fig. S1A) (20). Both Rbr and SOR require electron donors, usually a Rd and an NROR (Fig. S1B), but the partners of Rbr and Sor are still unknown in *C. difficile*. No genes encoding an isolated Rd are present in the *C. difficile* genome, and only two proteins, FdpF and CD0828, have a Rd domain. CD0828 is a protein of 480 amino acids, annotated in the *C. difficile* genome as a glutamate synthase. It harbors a Rd-like domain in its N-terminal part (residues 1–48), identified by the canonical motif C(xx)C(x)_21_C(xx)C. Another cysteine-rich sequence (residues 398–409) with a motif C(x)_5_C(x)_4_C is possibly indicative of the presence of an [FeS] cluster. Using Alphafold3 (35), we predicted a CD0828 structure model, which was then used as a template for the Alphafill server (36). The final model predicts the Rd domain, the presence of a [3Fe-4S]^1+/0^ cluster in the region mentioned above and a flavin mononucleotide (FMN) molecule as a cofactor for this protein (Fig. S2). The superposition of the obtained model with the crystallographic structure of a glutamate synthase from *Synechocystis sp. PCC 6803* (1498 amino acids) showed that CD0828 (480 amino acids) is putatively homologous to a portion of its C-terminal part (∼430 residues) (Fig. S2A) in line with the difference in length of the two polypeptides. The superimposable region contains the [3Fe-4S]^1+/0^ cluster and the FMN molecule (Fig. S2B). This representation also showed a small distance, of ~6.8 Å, between the FMN moiety and the [3Fe-4S]^1+/0^, which indicates that they are in the electron transfer range. Structural homology search in the FoldSeek server (37), using the model predicted for CD0828 as a template, identified several similar proteins. The model structure predicted for the putative glutamate synthase from *Methanocaldococcus jannaschii* (Uniprot Q58746) is the most similar (Fig. S2C), but homologous proteins were also identified in other bacteria such as *Pseudomonas aeruginosa* (Uniprot Q9HY24) or *Staphylococcus aureus* (Uniprot Q2FVF4). Contrary to canonical glutamate synthases, the length of these proteins is in the range of CD0828, with ca. 500 amino acids, and an identity range between 25% and 31%. The model structure of CD0828 also showed a loop of ~40 amino acids (residues 49–88) between the Rd and the glutamate synthase-like domains (Fig. S2A and C), which may indicate that the Rd domain has some degree of mobility. It is interesting to note that *CD0828* is the fourth gene of the *rbr* operon. Therefore, we could speculate its ability to donate electrons and be a partner of the Rbr and/or the Sor enzymes.

### Rbr, Sor, and CD0828 purification and redox properties

To characterize the role of Rbr and Sor proteins in ROS detoxification and the possible role of CD0828 as an electron transfer partner of these ROS reductases, we produced the proteins in *Escherichia coli* and purified them through several chromatographic steps. The molecular masses determined by SDS-PAGE were ∼21 kDa for Rbr, ∼12 and 25 kDa for Sor, and ~49 and 90 kDa for CD0828 in agreement with those calculated from their respective amino acid sequences, 20.6, 14, and 53 kDa, respectively (Fig. S3A through C). The existence of a mix of monomers and dimers for Sor and CD0828 was previously observed and could be explained by insufficient denaturating conditions. Size exclusion chromatography of the purified proteins revealed that Rbr is a tetramer in solution, with a molecular mass of ∼77 kDa (Fig. S3D) consistent with other Rbr structures (38). The Sor protein is mainly a dimer with a molecular mass of 32 kDa with a small contribution of a tetrameric form of ~63 kDa (Fig. S3E). Both arrangements were previously observed in solution for SOR and originate functional enzymes where 2Fe-SORs are usually dimers, whereas 1Fe-SORs are tetramers (20, 39, 40). CD0828 is present as a monomer and dimer, with molecular masses of 53 kDa and 106 kDa, respectively (Fig. S3F).

We then estimated the iron load of these purified proteins. For Rbr, the iron/protein monomer ratio was 1.6/1 instead of the expected 3/1 ratio (1 Fe atom in the Rd center and 2 Fe atoms in the diiron center), indicating a partially loaded enzyme, as observed for other proteins of this family (18). For Sor, a ratio of 2.3/monomer was obtained in accordance with the expected value for this protein (1 Fe atom in the center I, and another in the catalytic center II). CD0828 presented a ratio of 1.2/monomer, which is under the expected value assuming the iron of the Rd center and the expected [3Fe-4S]^1+/0^ cluster. The UV-visible spectrum of the oxidized Rbr was consistent with the presence of a Rd domain, with absorption maxima at 385 and 495 nm (Fig. S4A) (41, 42), whereas the spectrum of Sor was characteristic of a 2Fe-SOR in the “pink” semi-reduced state (center I in an oxidized state and center II in a reduced state) with maxima at 370 and 510 nm (Fig. S4B, dashed line). The fully oxidized spectrum presented an extra shoulder at 650 nm, characteristic of this oxidation state, “gray” form (Fig. S4B, solid line) (43). The spectral contribution of center II to the oxidized state spectrum was determined after subtracting the spectrum of the semi-reduced form from the fully oxidized one (Fig. S4B, dotted line). The UV-visible spectrum of CD0828 was also dominated by the contribution of the Rd center with maxima at 386 and 495 nm (Fig. S4C). The contribution of the [3Fe-4S]^1+/0^ center for the spectrum was difficult to detect, as these centers usually have broad absorption bands in the 300–500 nm region.

For the Rbr protein, the reduction potential of the Rd center could not be obtained by standard redox titrations monitored by visible spectroscopy in an anaerobic chamber at pH 7.5, as this center was already fully reduced after equilibration with the mediator’s mixture. Similar behavior was observed for the Sor Center II. However, we were able to determine the reduction potential of Center I. We obtained a value of ∼−120 ± 5 mV (Fig. S3G), which is much lower than the reduction potentials determined for previously isolated SORs, such as those from *Desulfovibrio desulfuricans* ATCC27774 or *Archaeoglobus fulgidus*, that are in the range of ∼+4 to ∼+60 mV, respectively (43, 44).

### Sor enzymatic activities

**Fig 1:**
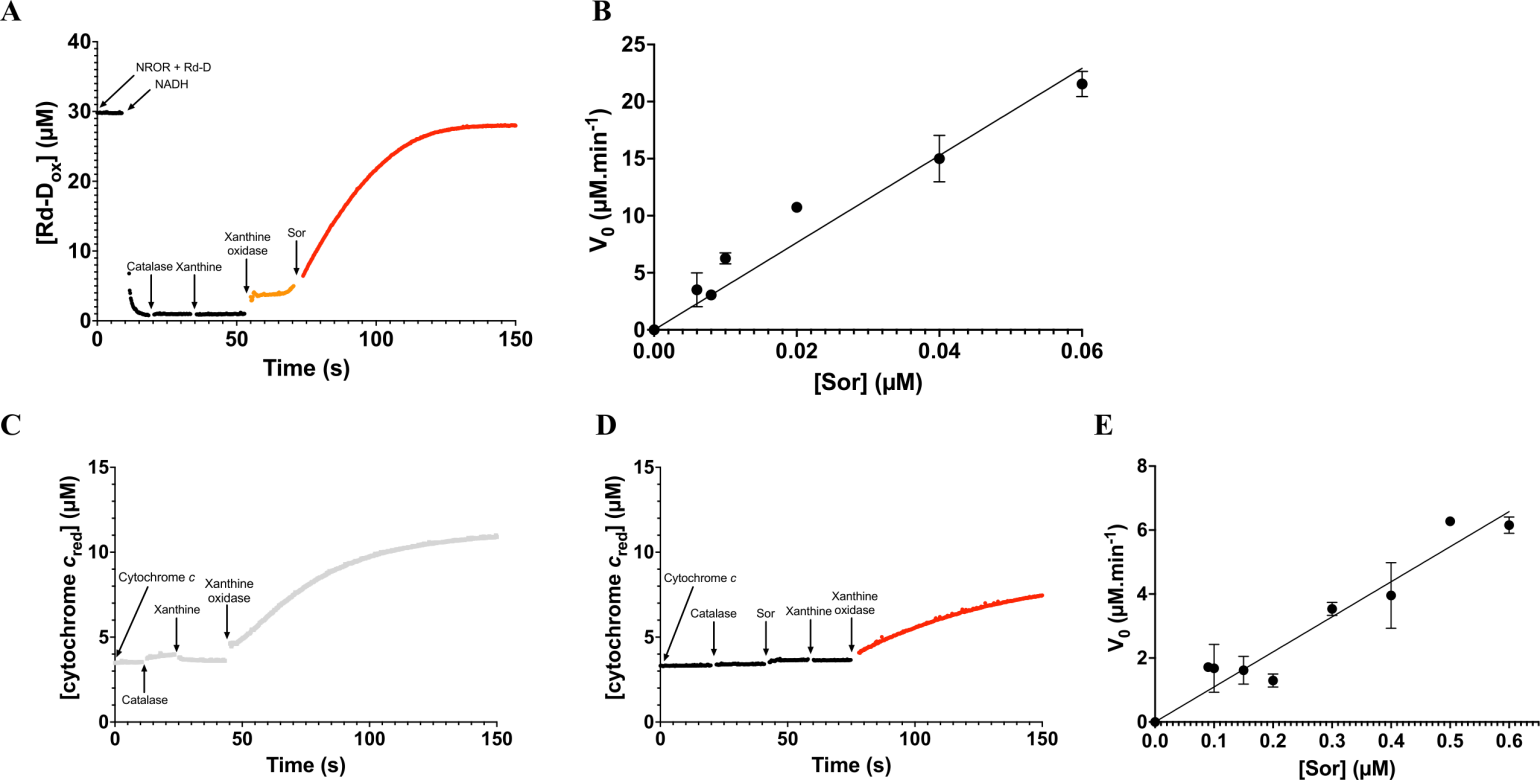
O_2_^•−^-reductase and O_2_^•−^-dismutase activities of the Sor protein. (**A**) The O_2_^•−^-reductase activity was determined in an air-saturated buffer by measuring Rd-D reoxidation rate monitored at 490 nm. The data presented are representative of the results obtained (at least three assays per concentration of Sor). Arrows indicate the time points of the successive additions of NROR (1 µM), Rd-D (30 µM), NADH (40 µM), catalase (640 nM), xanthine (1.5 mM), xanthine oxidase (0.046 units mL^−1^), and Sor. The black curve represents the reaction before the addition of the xanthine oxidase, leading to the continuous flux of O_2_^•−^. The orange curve corresponds to the start of the O_2_^•−^ flux before the addition of Sor (red curve). Experiments were performed in the presence of a 7.7 µM min^−1^ flux of O_2_^•−^ and different Sor concentrations ranging from 0.006 to 0.06 µM. (**B**) The O_2_^•−^-reductase activity rates (µM min^−1^) were plotted dependent on the Sor concentration, to calculate the apparent constant of the reaction, *k*_app_. (**C, D**) The O_2_^•−^-dismutase activity was determined in an air-saturated buffer by measuring cytochrome *c* reduction rate monitored at 550 nm. The data presented (at least three assays per concentration of Sor) are representative of the results obtained. Arrows indicate the time points of the successive additions of cytochrome *c* (10 µM), catalase (640 nM), xanthine (1.5 mM), xanthine oxidase (0.046 units mL^−1^), and Sor (panel D). Experiments were performed in the presence of a 7.7 µM min^−1^ flux of O_2_^•−^. (**C**) The gray curve represents the reaction without addition of the Sor. (**D**) The black curve represents the reaction before addition of the xanthine oxidase and the continuous flux of O_2_^•−^, whereas the red curve corresponds to the part with all the products added including Sor. Experiment was done with different Sor concentrations ranging from 0.09 to 0.6 µM. (**E**) The O_2_^•−^-dismutase activity rates (µM min^−1^) were plotted dependent on the Sor concentration to calculate the apparent constant of the reaction, *k*_app_.

Having established the presence of iron cofactors and determined the redox properties of Sor, we addressed the catalytic activities of the protein. Since the *C. difficile* physiological electron donors of this protein are still unknown, we used a heterologous system to perform the assays. We found that the truncated Rd domain (Rd-D) of *E. coli* flavodiiron protein (Fdp) and its associated reductase, homologous to NRORs, were able to reduce Sor in the presence of NADH. To test the O_2_^•−^-reductase activity, we spectrophotometrically measured the rate of reoxidation of Rd-D after the establishment of a continuous constant flow of O_2_^•−^ by the xanthine/xanthine oxidase system in a premix containing NADH, NROR, and Rd-D. This allowed for a slight reoxidation of the Rd-D, which was increased upon the addition of the Sor enzyme (Fig. 1A). A clear O_2_^•−^-reductase activity was observed in the presence of various concentrations of Sor (Fig. 1B). This allowed for the calculation of the *k*_app_ of the O_2_^•−^-reductase activity of 382 ± 8 min^−1^ (Fig. 1B; Table 1). This activity is about one order of magnitude larger than the one previously measured for the SOR from *Desulfovibrio vulgaris*, but in the same range as the one obtained for the SOR of *Treponema pallidum* (34).

**TABLE 1:** Enzymatic activities of the Sor and Rbr

Protein	Enzymatic activities	
Sor	O_2_^•−^-reductase activity (*k*_app_): 382 ± 8 min^−1^	O_2_^•−^-dismutase activity (*k*_app_): 11 ± 1 min^−1^
Rbr	H_2_O_2_-reductase activity: 1.4 ± 0.3 s^−1^	O_2_-reductase activity: 0.8 ± 0.1 s^−1^

As low O_2_^•−^-dismutase activities can also be detected in SOR enzymes (45–47), we measured this activity indirectly by following the decreased rate of reduction of horse heart cytochrome *c* after the establishment of a continuous constant flow of O_2_^•−^ by the xanthine/xanthine oxidase system in a solution containing oxidized cytochrome *c*. Without addition of Sor, we observed a rapid reduction of the cytochrome *c* (Fig. 1C). The addition of Sor diminished the rate of reduction of the cytochrome in a concentration-dependent manner (Fig. 1D). We thus observed a weak O_2_^•−^-dismutase activity, which is commonly observed for SOR proteins (47). This allowed for the calculation of the *k*_app_ of the O_2_^•−^-dismutase activity of 11 ± 1 min^−1^ (c.a. 30 U mg^−1^) (Fig. 1E; Table 1). This activity is in the same range as of O_2_^•−^-dismutase activities previously described for SORs of other organisms (45, 48).

### Rbr H_2_O_2_- and O_2_-reductase activities

**Fig 2:**
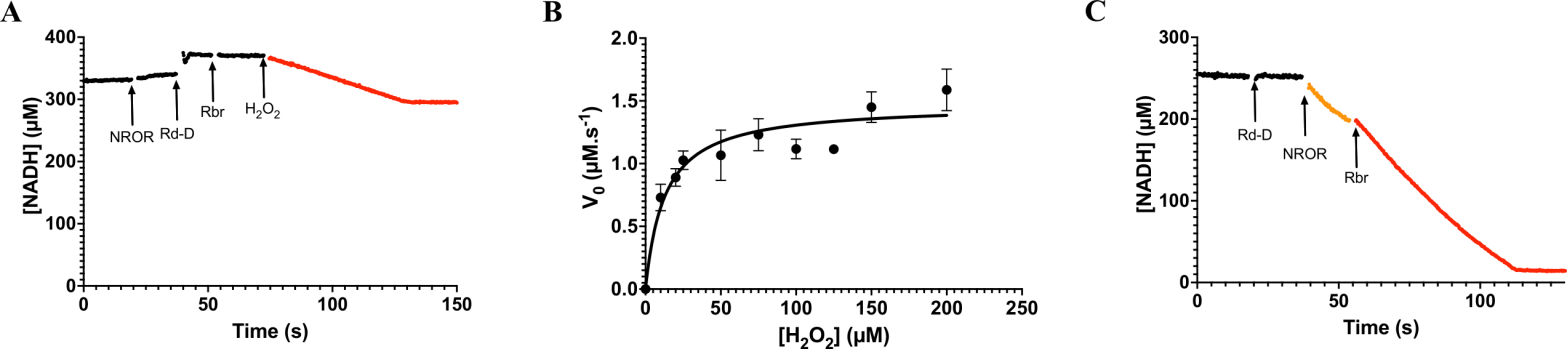
H_2_O_2_ and O_2_-reductase activities of the Rbr protein. (**A**) The H_2_O_2_ activity was determined anaerobically by measuring NADH consumption monitored at 340 nm. The data presented are representative of the results obtained (at least three assays per concentration of H_2_O_2_). Arrows indicate the time points of the successive additions of NROR (0.4 µM), Rd-D (2 µM), Rbr (1 µM), and H_2_O_2_. The black curve represents the reaction before the addition of H_2_O_2_. The red curve corresponds to the part with all the products added. Experiments were performed in the presence of 200 µM NADH and different H_2_O_2_ concentrations ranging from 10 to 200 µM. The H_2_O_2_-reductase activity rate (s^−1^) from Table 1 is the result of the subtraction of the experimental slope (µM/s) before and after the addition (in red) of the enzyme divided by the protein concentration (µM). (**B**) The H_2_O_2_-reductase activity rates (µM s^−1^) resulted from the subtraction of the experimental slope (µM/s) before and after the addition of H_2_O_2_. The rates were plotted dependent on the H_2_O_2_ concentration to produce a Michaelis-Menten plot. (**C**) The O_2_-reductase activity was determined in air-saturated buffer (around 260 µM O_2_) by measuring NADH consumption monitored at 340 nm. The data presented are representative of the results obtained (at least five assays). Arrows indicate the time points of the successive additions of Rd-D (2 µM), NROR (0.4 µM), and Rbr (2 µM). The black curve represents the reaction before the addition of NROR and Rbr. The orange curve corresponds to the part before the addition of the Rbr allowing the stabilization of the NROR O_2_ reduction. The red curve corresponds to the part with all the products added. Experiments were performed in the presence of 200 µM NADH. The O_2_-reductase activity rate (s^−1^) from Table 1 is the result of the subtraction of the experimental slope (µM s^-1^) before (in orange) and after the addition (in red) of the enzyme divided by the protein concentration (µM).

The Rbr proteins are H_2_O_2_-reductases but an O_2_-reductase activity can be also observed (21). The two activities were determined using NADH as the primary electron donor. As expected, Rbr alone did not show any NADH oxidase activity before the addition of H_2_O_2_ (Fig. 2A). In the absence of known electron donors in *C. difficile,* we used the system utilized for Sor to reduce Rbr in the presence of NADH. We observed an H_2_O_2_-reductase activity with a rate of 1.4 ± 0.3 s^−1^ (Fig. 2B; Table 1). This activity is rather similar to that previously described for the revRbr of *C. acetobutylicum* and the two revRbrs and the FdpF of *C. difficile* (18, 21, 25). Additional assays were performed with different concentrations of H_2_O_2_. The dependence of the reaction rates with the substrate concentration revealed that this enzyme had a Michalis-Menten behavior, allowing us to estimate a *K_m_* of 13 ± 3 µM and a *V_max_* of 1.5 ± 0.1 µM s^−1^ (Fig. 2B). The O_2_-reductase activity was measured by the same method. Rbr was added to an air-saturated buffer (∼260 µM O_2_) containing NADH as well as a NROR and a Rd-D from *E. coli*. We observed an O_2_-reductase activity with rates of 0.8 ± 0.1 s^−1^ (Fig. 2C; Table 1). This activity is slightly lower than that observed for the other O_2_-reducing enzymes of *C. difficile* (18). Nevertheless, these activities of Rbr may be underestimated since the physiological partners in *C. difficile* remain to be identified. We can conclude that Rbr acts both as NADH-linked H_2_O_2_- and O_2_-reductases *in vitro*.

### Is CD0828 a potential electron donor for Rbr and Sor?

**Fig 3:**
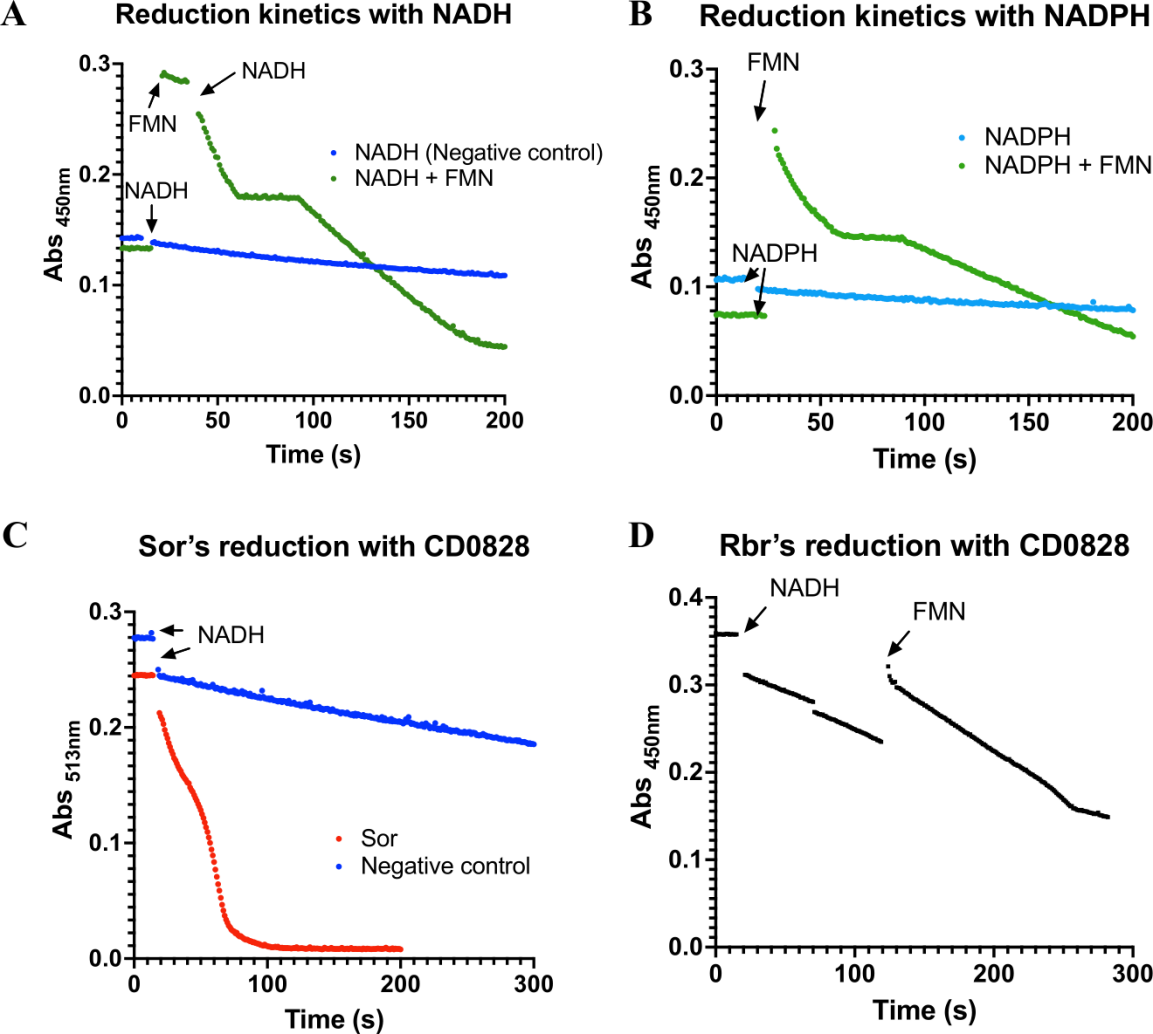
CD0828 reduction with NADH or NADPH in the presence or absence of FMN, Sor, and Rbr. CD0828 reduction by NAD(P)H was evaluated anaerobically, inside an anaerobic chamber. (**A**) CD0828 was incubated only with NADH (blue trace) or with NADH and FMN (green trace). (**B**) CD0828 was incubated only with NADPH (blue trace) and with NADPH and FMN (green trace). The reaction was monitored by following the reduction of CD0828 (and FMN) at 450 nm. Reagents’ concentrations in the assay were 20 µM of CD0828, 200 µM NADH or NADPH, and 40 µM FMN in 100 mM MOPS, pH 7.5, and 150 mM NaCl. (**C**) The electron transfer between CD0828 and Sor was evaluated by monitoring the reduction of Sor’s center I enzyme, at 513 nm (orange trace). The control represents the Sor reduction only with NADH (blue trace). Reagent’s concentrations in the assay were 30 µM of Sor, 10 µM of CD0828, and 20 µM FMN and 200 µM NADH in 100 mM MOPS, pH 7.5, and 150 mM NaCl. (**D**) The electron transfer between CD0828 and Rbr was monitored at 450 nm (black trace). Reagent’s concentrations in the assay were 40 µM of Rbr, 10 µM of CD0828, 20 µM FMN, and 200 µM NADH in 100 mM MOPS, pH 7.5, and 150 mM NaCl. The assays are representative of at least triplicates.

To evaluate the possibility that CD0828 can act as a reductase for both Rbr and Sor, its own ability to receive electrons directly from NAD(P)H was first investigated. Successive additions of sub-stoichiometric amounts of NADH or NADPH were performed under anaerobic conditions, and the reduction of CD0828 was followed by UV-visible spectroscopy. These assays showed that purified CD0828 can be completely reduced by NADH but not NADPH (Fig. S4D and E). Then, the rate of reduction with both electron donors was monitored at 450 nm in the presence or absence of FMN to evaluate its role in the electron transfer. The data confirmed that the CD0828 reduction with NADH occurs spontaneously, despite with a slow rate (Fig. 3A), but not with NADPH (Fig. 3B). The addition to the reaction mixture of FMN, which is the usual cofactor in similar proteins, enabled the electron transfer from NADPH to CD0828 (Fig. 3B) and increased the NADH electron transfer rate (Fig. 3A). This indicated that CD0828 requires the presence of FMN for the redox reaction, resulting in a fully reduced protein. These assays also indicated that NADH is the most effective electron donor.

The capacity of CD0828 to reduce Sor was monitored by UV-visible spectroscopy at 513 nm, following the reduction of center I. Sor was incubated with CD0828 in the presence of NADH and FMN (Fig. 3C). Sor’s reduction occurs at two different rates. The first one, taking about 30 s, is consistent with the FMN’s reduction when compared with the previous assays. The second corresponds to the Sor’s center I reduction, which needs about 25 s to get fully reduced. The capacity of CD0828 to reduce Rbr was also tested by UV-visible spectroscopy at 450 nm (Fig. 3D), monitoring the reduction of the Rd center. The process was even slower than with the Sor, as 200 s were necessary to become almost fully reduced. We cannot exclude that the FMN supplementation is not sufficient to achieve the best conditions for the electron transfer between CD0828 and Sor or Rbr. As the reduction rates observed in these experiments are low, it seems unlikely that CD0828 acts as an efficient electron donor for Sor or Rbr *in vivo*.

### Defense enzymes contributing to ROS tolerance in *C. difficile*

**Fig 4:**
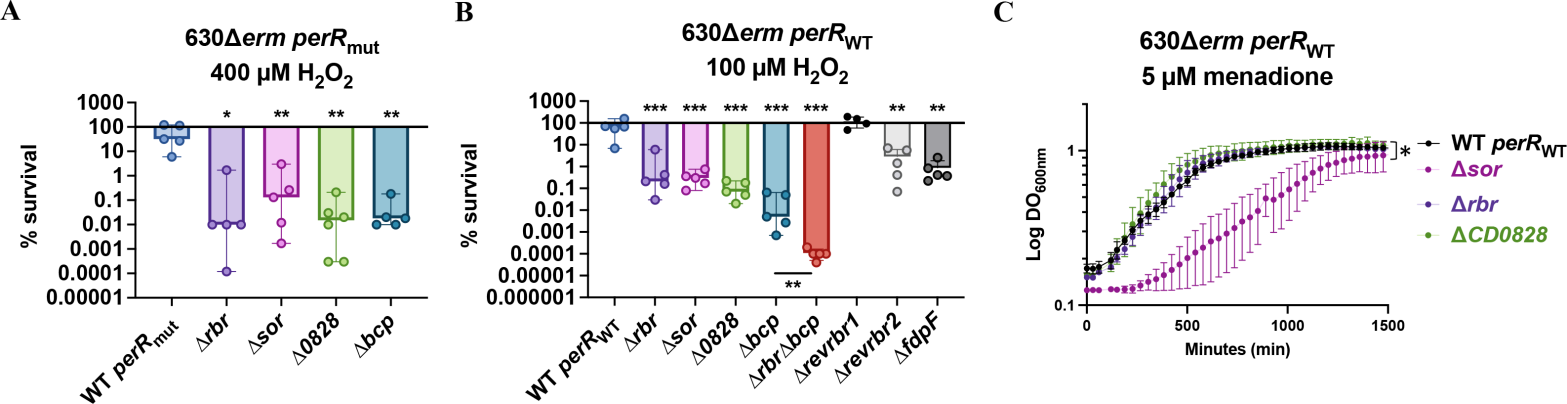
Role of genes of the *perR* operon and of *bcp* in H_2_O_2_ and menadione resistance. (**A, B**) WT, single mutants inactivated for *rbr*, *sor*, *CD0828*, or *bcp* in *perR*_mut_ strain (**A**) and *perR*_WT_ strain (**B**) were tested. The double ∆*rbr* ∆*bcp* mutant and the *revrbr1*, *revrbr2*, and *fdpF* mutants of the 630∆*erm perR*_WT_ strain were also tested (**B**). These strains were exposed for 30 min to 400 µM (A, *perR*_mut_) or 100 µM H_2_O_2_ (B, *perR*_WT_) in a glycylglycine buffer. Serial dilutions were spread in BHI plates that were incubated in anaerobiosis for 24 h. CFUs were determined before and after H_2_O_2_ exposure, and the ratio was calculated to obtain the percentage of survival. Five biological replicates were performed per experiment. For all histograms, the mean with standard deviation (SD) are shown. Ordinary one-way ANOVA was performed followed by Dunett’s multiple comparison test with the 630∆*erm* strain *perR*_mut_ or *perR*_WT_. Mann-Whitney statistical test was performed for ∆*bcp* and ∆*rbr* ∆*bcp* comparison *: *P*-value < 0.05 ; **: *P*-value < 0.01. (**C**) 630∆*erm perR*_WT_ strain and single mutants inactivated for *rbr*, *sor,* or *CD0828* were dispatched in 96-well plates containing 5 µM of menadione. The OD_600_ was followed for 24 h in a plate reader under anaerobiosis conditions. The mean of four independent experiments and standard deviation (SD) are represented. Two-way ANOVA was performed between ∆*sor* and WT. *: *P*-value < 0.05

Sor and Rbr have, respectively, O_2_^•−^- and H_2_O_2_-reductase activities *in vitro,* and the possible role of CD0828 in ROS response remains to be clarified. To determine the contribution of the genes of the *rbr* operon to the resistance to ROS, we constructed mutants deleted for each gene (*rbr*, *sor*, or *CD0828*) in the 630∆*erm perR*_mut_ and the 630∆*erm perR*_WT_ strains. Then, we tested the survival of the different mutants after 30 min exposure to H_2_O_2_. As the T41A mutation present in *perR*_mut_ is known to lead to a derepression of the *rbr* operon (27), we tested several concentrations of H_2_O_2_ to optimize the conditions for the *perR*_WT_ and the *perR*_mut_ strains. Two different concentrations of H_2_O_2_ were chosen, 400 µM for the 630∆*erm perR*_mut_ strain and 100 µM for the 630∆*erm perR*_WT_ strain. After 30 min exposure to H_2_O_2_ at these concentrations, a survival between 50% and 100% was observed for both strains. As expected from previously observed survival in air (27), this result indicates that the *perR*_mut_ strain is more resistant to H_2_O_2_ than the *perR*_WT_ strain. In the two different *perR* backgrounds, we observed a drastic reduction of survival between 3- and 5-log in all the mutants compared with the parental strains (Fig. 4A and B). These phenotypes were fully or partially complemented by plasmids carrying the *rbr* or the *CD0828* gene, while we complemented the *sor* mutant in the *perR*_WT_ strain (Fig. S5A and B). These results indicate a physiologically relevant role of *rbr*, *sor*, and *CD0828* in H_2_O_2_ resistance. Rbr is directly involved in H_2_O_2_ detoxification, whereas CD0828 could play a role in ROS response by a mechanism either independent or dependent on Rbr. The more complex possible involvement of Sor will be addressed in the discussion section.

In *C. difficile,* a Prx, Bcp (CD1822), could also contribute to H_2_O_2_ detoxification. We constructed a single ∆*bcp* mutant in the two *perR* backgrounds and a double ∆*rbr* ∆*bcp* mutant in the *perR*_WT_ strain. We observed a drastic reduction in survival of the ∆*bcp* mutant compared with the parental strain in both the *perR*_mut_ and the *perR*_WT_ backgrounds (Fig. 4A and B). The defect in survival was even slightly more important in the ∆*bcp* mutant than in the ∆*rbr* mutant in the *perR*_WT_ strain. In addition, upon exposure to H_2_O_2_, the double ∆*rbr* ∆*bcp* mutant of the strain 630∆*erm perR*_WT_ survived significantly less than the single mutants (Fig. 4B). These results indicate that Bcp plays a crucial role in resistance to H_2_O_2_ even in a *perR*_mut_ strain overproducing Rbr. The involvement of Bcp is likely due to H_2_O_2_ detoxification through its peroxidase activity.

Finally, we have previously shown that FdpF and the two revRbrs have an H_2_O_2_-reductase activity *in vitro* (18, 25). However, no difference in H_2_O_2_ susceptibility was detected for the triple ∆*revrbr*1/2 ∆*fdpF* mutant in a disc diffusion assay compared with the parental *perR*_mut_ strain. As this test has been done in the 630∆*erm perR*_mut_ background, we hypothesized that the overexpression of the *rbr* operon may mask the impact of the inactivation of *revrbr1*, *revrbr2,* or *fdpF*. Hence, we deleted these three genes in the 630∆*erm perR*_WT_ strain. In this strain, the ∆*fdpF* and ∆*revrbr2* mutants, but not the ∆*revrbr1* mutant, have a significant defect in survival upon H_2_O_2_ exposure compared with the parental strain (Fig. 4B). This effect is less severe than the drop in survival observed in the ∆*bcp* or the ∆*rbr* mutants, suggesting that FdpF and revRbr2 have a more minor role in H_2_O_2_ detoxification compared with Rbr and Bcp. All these results indicate that *C. difficile* has an arsenal of enzymes to counteract H_2_O_2_ exposure.

To address the possible role not only of the O_2_^•−^-reductase, Sor, but also of the other enzymes encoded by the *rbr* operon in O_2_^•−^ detoxification, we exposed the mutants and the parental *perR*_WT_ strain to menadione, a soluble naphtoquinone known to generate high amounts of O_2_^•−^ in the presence of O_2_ (49, 50) (Fig. 4C). We observed a drastic growth defect of the ∆*sor* mutant compared with the parental *perR*_WT_ strain in the presence of 5 µM of menadione. This result confirms the specific role of Sor in the detoxification of O_2_^•−^ as recently published in a RT027 strain (26). By contrast, the ∆*rbr* mutant was not affected in these conditions. As O_2_^•−^ reduction produces H_2_O_2_, the absence of phenotype of the ∆*rbr* mutant suggests that Bcp, revRbr2, and FdpF seem to be sufficient to detoxify the H_2_O_2_ produced. In addition, the ∆*CD0828* mutant had the phenotype of the parental *perR*_WT_ strain. The absence of phenotype of the ∆*CD0828* mutant in the presence of menadione suggests that CD0828 is probably not involved in the elimination of O_2_^•−^ or in the electron transfer to Sor, in agreement with the low electron transfer efficiency between CD0828 and Sor *in vitro* (Fig. 3C).

### Role of ROS detoxification enzymes in *C. difficile* survival to air exposure

**Fig 5:**
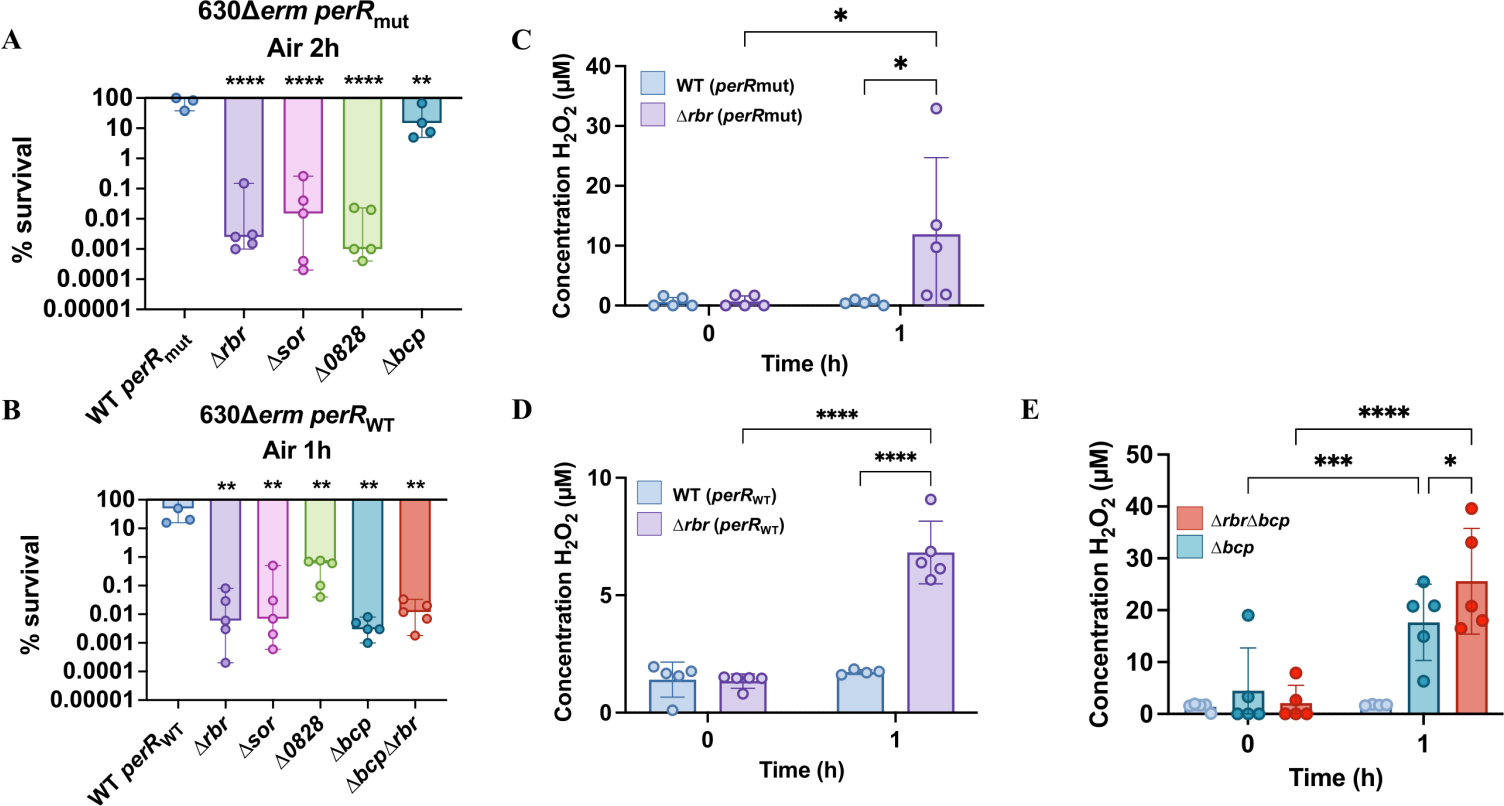
Phenotype of mutants of genes encoding ROS detoxification enzymes and H_2_O_2_ production after air exposure. (**A, B**) Serial dilutions of the WT and single mutants inactivated for *rbr*, *sor*, *CD0828,* or *bcp* in *perR*_mut_ (**A**) and *perR*_WT_ (**B**) backgrounds as well as the double ∆*rbr* ∆*bcp* mutant and the *revrbr1*, *revrbr2*, and *fdpF* mutants of the 630∆*erm perR*_WT_ strain (**B**) were spotted on TY Tau plates. Plates were incubated either in anaerobiosis for 24 h or in presence of air during 2 h (A, *perR*_mut_) or 1 h (B, *perR*_WT_), followed by an incubation of 24 h in anaerobiosis. CFUs were determined for each experiment, and the percentage of survival after air exposure relative to the survival in anaerobiosis was plotted. Ordinary one-way ANOVA was performed followed by Dunett’s multiple comparison test with the 630∆*erm* strain *perR*_mut_ or *perR*_WT_. *: *P*-value < 0.05; **: *P*-value < 0.01. (**C, D**) Measurements of H_2_O_2_ concentration (µM) in supernatants of the *perR*_mut_ (**C**) and the *perR*_WT_ (**D**) backgrounds in glycylglycine buffer with or without 1 h of air exposure. The parental strains are indicated in light blue and the isogenic single ∆*rbr* mutant in purple. (**E**) Measurements of H_2_O_2_ concentration (µM) in the supernatants of the 630∆*erm perR*_WT_ (light blue), the single ∆*bcp* (dark blue) and the double ∆*rbr* ∆*bcp* (red) mutants with or without 1 h of air exposure. The mean of 5 independent experiments and standard deviation are represented. Two-way ANOVA was performed. *: *P*-value < 0.05 ; **: *P*-value < 0.01.

Although *C. difficile* is considered as an obligate anaerobe, this bacterium can survive exposure to high O_2_ tension and even to air (32, 51, 52). We have previously determined the contribution of the different O_2_-reductases of *C. difficile* in air tolerance (15). Hence, we focused our study on the contribution of Rbr, Sor, CD0828, and Bcp in the survival of *C. difficile* in the presence of air. After optimization of the time of exposure for each background, serial dilutions of cultures of the different mutants on plates were incubated in air for 1 h or 2 h for the *perR*_WT_ and the *perR*_mut_ strains, respectively, followed by a 24 h incubation in anaerobiosis. We showed a drastic decrease in survival for all mutants in the *perR*_WT_ background. In the *perR*_mut_ strain, an important decrease in survival (3–5 log) was observed except for the ∆*bcp* mutant, which is significantly less affected compared with the other mutants with only a 10-fold decrease in survival (Fig. 5A and B). The survival of complemented strains was partly or fully restored (Fig. S5C). These results indicated a significant role of Rbr, Sor, CD0828, and Bcp during exposure of *C. difficile* to air. After SlpA, Rbr is the second most abundant protein found in the strain 630∆*erm perR*_mut_ (53). This might explain why the phenotype of the ∆*bcp* mutant in air is more drastic in the *perR*_WT_ than in the *perR*_mut_ background. Indeed, the overproduction of Rbr probably overshadows the impact of *bcp* inactivation. Our results are also consistent with those obtained in a recent study showing that a *sor::erm* mutant had a defect of survival after exposure to air in a RT027 strain (26).

To confirm endogenous ROS production upon air exposure, we used a colorimetric kit to detect H_2_O_2_. We showed an increase in H_2_O_2_ concentration in the supernatant after 1 h of air exposure for the ∆*rbr* mutant compared with the parental strain in both the *perR*_mut_ and the *perR*_WT_ backgrounds (Fig. 5C and D), suggesting an accumulation of endogenous H_2_O_2_ in the absence of the Rbr peroxidase. Using the same approach, we also tested H_2_O_2_ production in the ∆*bcp* and the double ∆*bcp*∆*rbr* mutant in the *perR*_WT_ strain (Fig. 5E). After 1 h in air, we observed an accumulation of H_2_O_2_ in the supernatant of the ∆*bcp* mutant with a production even higher than in the ∆*rbr* mutant. In addition, we observed a cumulative effect in the double ∆*bcp* ∆*rbr* mutant, illustrating the contribution of both Bcp and Rbr in H_2_O_2_ detoxification. We therefore confirmed the production of endogenous H_2_O_2_ by the tested strains upon air exposure, which is associated with their impaired H_2_O_2_ detoxification.

### Role of Rbr, Sor, CD0828, and Bcp in the protection of *C. difficile* to physiological O_2_ tensions

**Fig 6:**
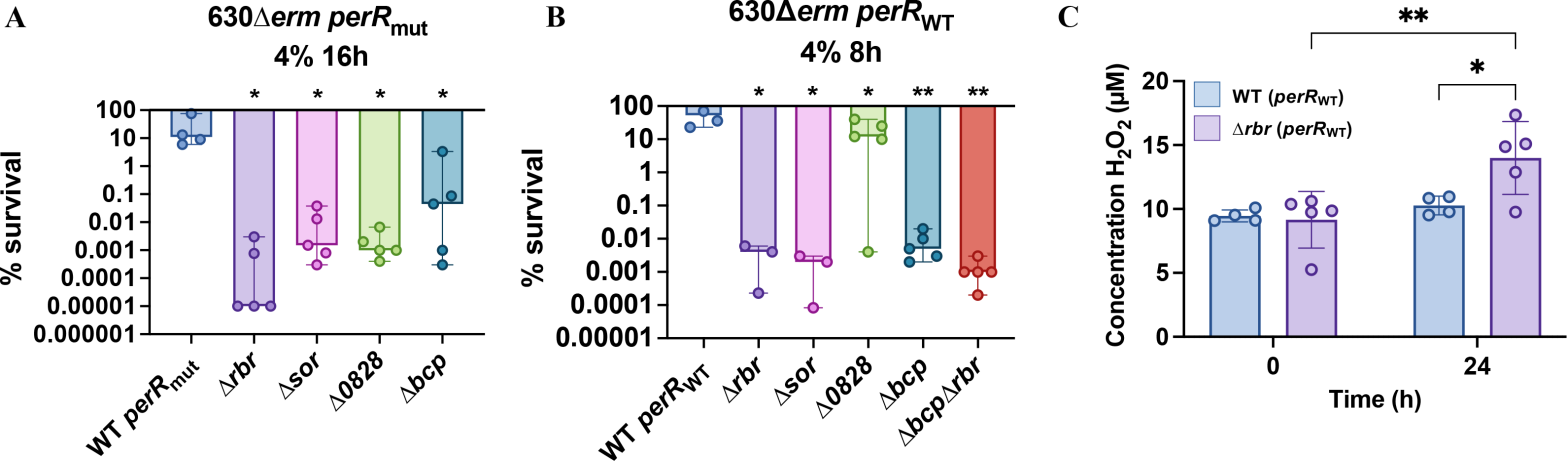
Phenotype of mutants of genes encoding ROS detoxification enzymes and H_2_O_2_ production after 4% O_2_ exposure. (**A, B**) Serial dilutions of the WT and single mutants inactivated for *rbr*, *sor*, *CD0828,* or *bcp* in *perR*_mut_ (**A**) and *perR*_WT_ (**B**) backgrounds and the double ∆*rbr* ∆*bcp* mutant of the 630∆*erm perR*_WT_ strain (**B**) were spotted on TY Tau plates. Plates were incubated in 4% O_2_ for 16 h or 8 h, for 630∆*erm perR*_mut_ and 630∆*erm perR*_WT_, respectively, followed by 24 h in anaerobiosis before CFUs counting. At least four biological replicates were performed per experiment. For all plots, mean with standard deviation is shown. Ordinary one-way ANOVA was performed followed by Dunett’s multiple comparison test with the 630∆*erm* strain *perR*_mut_ or *perR*_WT_. *: *P*-value < 0.05 ; **: *P*-value < 0.01. (**C**) Measurements of H_2_O_2_ concentration (µM) in supernatants of the 630∆*erm perR*_WT_ (light blue) and the ∆*rbr* mutant (purple) in glycylglycine buffer after 24 h at 4% O_2_ or in anaerobiosis. The mean of at least four independent experiments and standard deviation (SD) are represented. Two-way ANOVA was performed. *: *P*-value < 0.05 ; **: *P*-value < 0.01.

We also evaluated the involvement of both the *rbr* operon and the *bcp* gene in the survival of *C. difficile* and the endogenous H_2_O_2_ production after exposure to physiological O_2_ tensions. At 1% O_2_, a tension encountered near the mucus layer in an inflamed colon during CDI (54), we showed no difference in the survival of the *perR*_mut_ and *perR*_WT_ strains and no difference either in the single mutants (Fig. S6A and B). The absence of phenotype at this O_2_ tension is correlated with an absence of H_2_O_2_ detected in the supernatant under these conditions (Fig. S6C). At 4% O_2_, a tension encountered during spore germination in the small intestine (55), we observed a drastic reduction in the survival of the ∆*rbr*, ∆*sor,* and ∆*bcp* mutants in the *perR*_mut_ and the *perR*_WT_ backgrounds after 16 h or 8 h, respectively. A drastic survival defect was also observed for the double ∆*rbr* ∆*bcp* mutant (*perR*_WT_) (Fig. 6A and B). The survival of the complemented strains was partly or fully restored except for *CD0828* in the *perR*_WT_ background (Fig. S5D and E). We then measured the H_2_O_2_ concentration in the medium after 24 h exposure to 4% O_2_ for the *perR*_WT_ strain and the ∆*rbr* mutant. We showed a significant increase in H_2_O_2_ concentration in the mutant compared to the parental strain (Fig. 6C). These data indicate that *C. difficile* produces endogenous H_2_O_2_ upon O_2_ exposure at a tension ≥4%.

### Induction of the genes encoding ROS detoxification systems by H_2_O_2_ and air and their control by PerR

**Fig 7:**
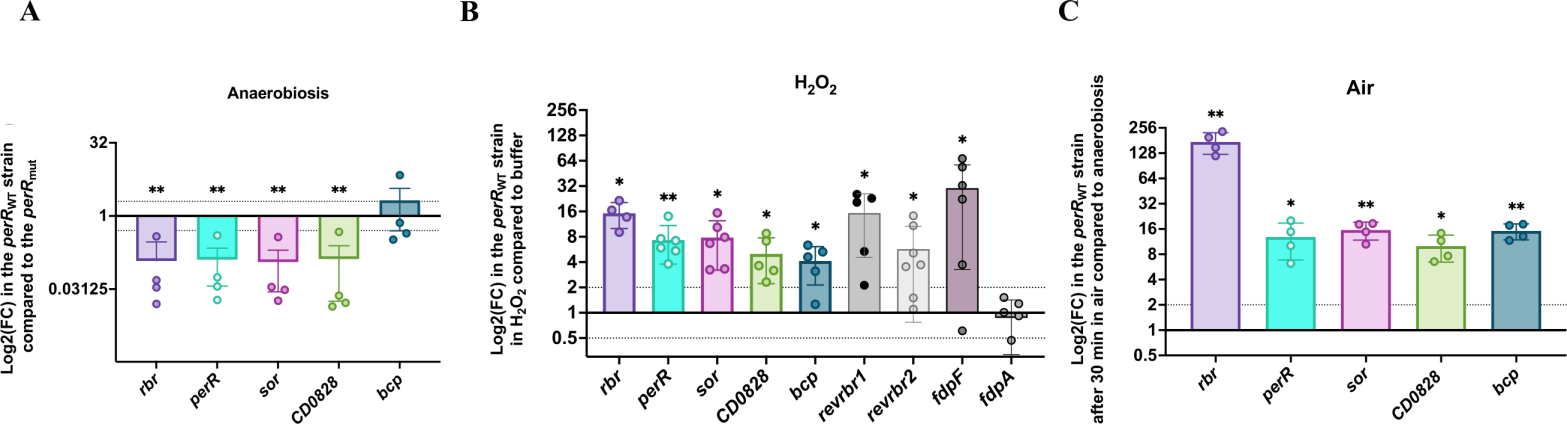
Expression of the genes encoding ROS-detoxification enzymes upon exposure to H_2_O_2_ or air. Differential expression of the *rbr*, *perR*, *sor*, *CD0828, bcp*, *revrbr1*, *revrbr2*, *fdpF,* and/or *fdpA* genes was evaluated by RT-qPCR. We compared the expression of 630∆*erm perR*_WT_ and 630∆*erm perR*_mut_ after 24 h in TY in anaerobiosis for the *rbr*, *perR*, *sor*, *CD0828,* and *bcp* genes (**A**), of the 630∆*erm perR*_WT_ without or with 30 min exposure to 100 µM H_2_O_2_ in glycylglycine buffer for all the genes (**B**) or of the 630∆*erm perR*_WT_ without or with 30 min of air exposure in TY for the *rbr*, *perR*, *sor*, *CD0828,* and *bcp* genes (**C**). *pgi* or *gyrA* were used as reference genes. Experiments were performed on at least four biological replicates. Mean and standard deviation (SD) are shown. One sample *t*-tests were used with a comparison of the fold change to 1. ns: not significant, *: *P*-value < 0.05; **: *P*-value < 0.01.

**Fig 8:**
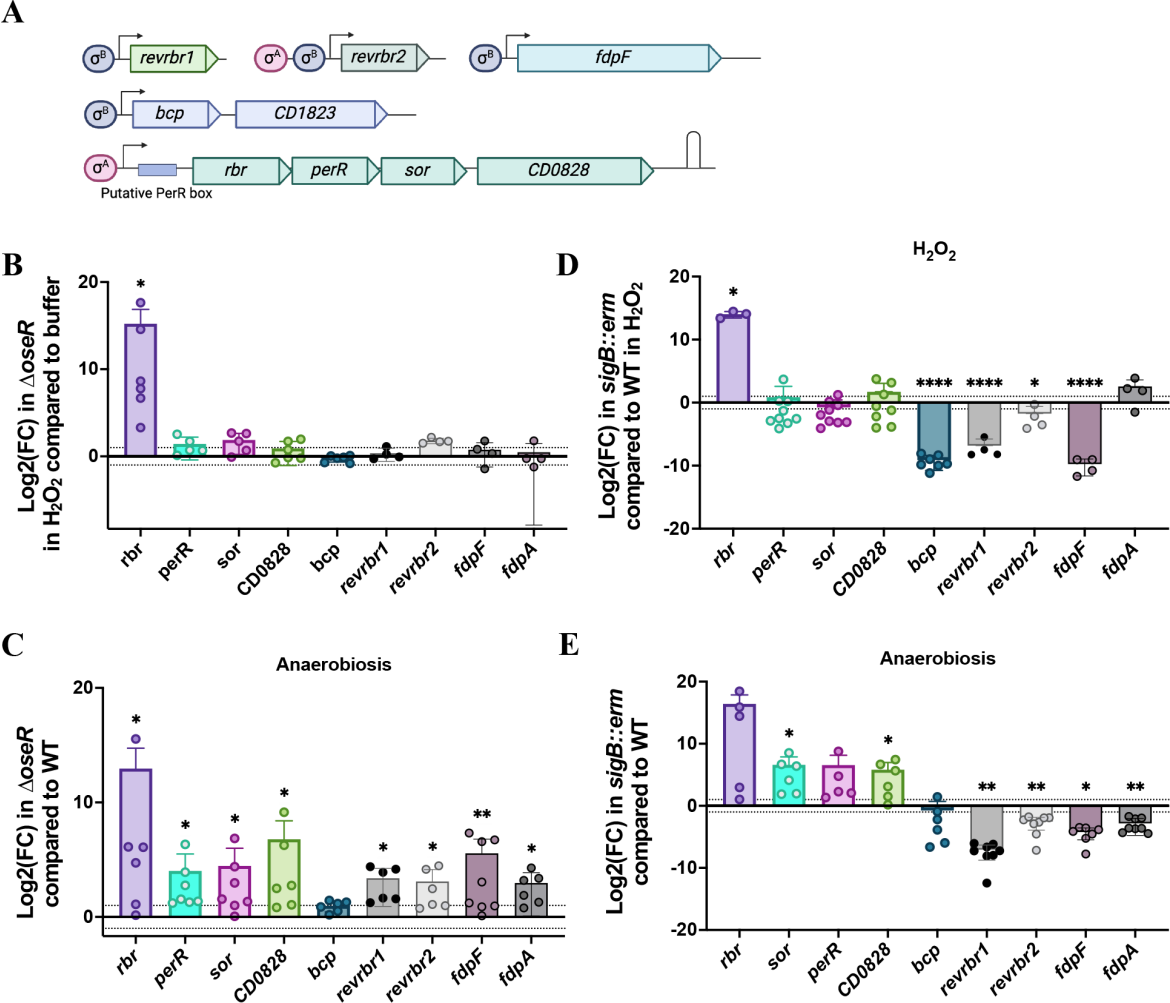
Regulation of the genes encoding ROS-detoxification under H_2_O_2_ and air stress. (**A**) Schematic representation of each operon. (**B–E**) Differential expression of the *rbr*, *perR*, *sor*, *CD0828*, *bcp, revrbr1*, *revrbr2*, *fdpF,* and *fdpA* genes was evaluated by RT-qPCR represented in log2(Fold Change). We compared the expression of the *perR*_WT_*∆oseR* mutant after 30 min in the presence or absence of 100 µM H_2_O_2_. (**B**) The *∆oseR* mutant and the *perR*_WT_ strain in anaerobiosis. (**C**) The *perR*_WT_ *sigB::erm* mutant and the *perR*_WT_ strain after 30 min in the presence of 100 µM H_2_O_2_, (**D**) and the *sigB::erm* mutant and the *perR*_WT_ strain in anaerobiosis. (**E**) *pgi* or *gyrA* were used as reference genes. Experiments were performed on at least four biological replicates. Mean and standard deviation (SD) are shown. One sample *t*-tests were used with a comparison of the fold change to 1. ns: not significant, *: *P*-value < 0.05; **: *P*-value < 0.01.

We then wanted to determine if the genes encoding the enzymes involved in ROS detoxification are similarly regulated. The *bcp* gene forms an operon with another gene *CD1823* encoding a protein of unknown function. The *rbr*, *perR*, *sor,* and *CD0828* genes belong to the same operon controlled by PerR (see Fig. 8A) (27). By comparing the expression of all these genes in the *perR*_WT_ and the *perR*_mut_ strains grown in anaerobiosis for 24 h, we were able to demonstrate a significant derepression of the genes of the *rbr* operon in the *perR*_mut_ strain compared with the *perR*_WT_ strain but not of the *bcp* gene (Fig. 7A). These results confirm that PerR negatively controls the expression of its own operon (27) and indicate that the *bcp* gene is not controlled by PerR as previously observed for the *revrbr* and the *fdp* genes (15).

To avoid the effects of the mutation in the *perR* gene, we then investigated the differential expression of the genes encoding enzymes involved in ROS resistance only for the *perR*_WT_ strain. We showed that 30 min of exposure to H_2_O_2_ or to air of the strain 630∆*erm perR*_WT_ led to a significant increase of expression of the *rbr*, *perR*, *sor*, *CD0828*, and *bcp* genes, compared with conditions without stress (Fig. 7B and C). In air, the production of endogenous H_2_O_2_ (Fig. 5D) could suggest that the induction observed might be due to an H_2_O_2_-dependent control. In addition, the expression of the *revrbr* and the *fdp* genes also increased following air exposure (Fig. S7A) as previously observed in the *perR*_mut_ strain (15). We also observed an induction of the expression of the *revrbr1*, *revrbr2*, and *fdpF* upon H_2_O_2_ exposure (Fig. 7B). By contrast, the expression of *fdpA* is not induced by H_2_O_2_, indicating a differential regulation. Only the genes encoding O_2_-reductases with an H_2_O_2_-reductase activity *in vitro*, the two revRbr and FdpF (18, 25), are thus induced by H_2_O_2_.

### Regulation of the *rbr* operon and the *bcp* gene by OseR

We recently identified an O_2_-responsive regulator belonging to the Spx family, OseR, which has been characterized as a regulator of the *fdp* and *revrbr* genes encoding the O_2_-reducing enzymes (15). OseR is involved in the control of these genes during long-term exposure to 1% O_2_ (15). We therefore wondered whether OseR could be also involved in the H_2_O_2_ or air-dependent induction of these genes. We showed that the induction of the expression of the *revrbr1*, *revrbr2,* and *fdpF* genes by H_2_O_2_ (Fig. 7B) disappeared in the ∆*oseR* mutant (Fig. 8B), whereas we confirmed the derepression of these three genes in the ∆*oseR* mutant in anaerobiosis (Fig. 8C). The same pattern was observed after exposure to air (Fig. S7B). We then compared the impact of *oseR* inactivation on the expression of the *bcp*, *rbr*, *perR*, *sor*, and *CD0828* genes in anaerobiosis, H_2_O_2_ and air (Fig. 8B and C; Fig. S7B and C). In anaerobiosis, we observed a derepression of all the genes belonging to the *rbr* operon in the ∆*oseR* mutant compared with the *perR*_WT_ strain, whereas the *bcp* gene is not differentially expressed (Fig. 8C). In the presence of H_2_O_2_ or air, the repression by OseR of *perR*, *sor*, and *CD0828* disappeared (Fig. 8B; Fig. S7B and C). Interestingly, the induction of the expression of the *rbr* gene by H_2_O_2_ was still high in the ∆*oseR* mutant (Fig. S7C). This must be explained by an additional level of regulation by H_2_O_2_ specific to this gene. These results confirm that OseR is involved in the regulation of the *rbr* operon but does not control *bcp* expression in the conditions tested. In addition, the ∆*oseR* mutant did not show any loss of survival compared with *perR*_WT_ strain when exposed to air for 1 h (Fig. S7D).

### Role of σ^B^ in the control of the genes encoding ROS detoxification systems

As the induction of *bcp* is independent of PerR and OseR, we wanted to identify other factors involved. Analysis of a genome-wide transcription start site (TSS) mapping (56) indicates the presence of a consensus recognized by σ^B^ upstream of the TSS of the *bcp-CD1823* operon (Fig. 8A). We thus tested the potential role of σ^B^, the sigma factor of the stress response. By RT-qPCR, we compared the expression of the *bcp* gene in the *sigB::erm* mutant, and the parental *perR*_WT_ strain in the absence or presence of stress exposure (30 min in H_2_O_2_ or air). We observed a drastic decrease in expression of the *bcp* gene in the *sigB::erm* mutant upon stress exposure but not in anaerobiosis (Fig. 8D and E; Fig. S7E). The presence of a consensus recognized by σ^B^ upstream of the TSS of *bcp* and the strong impact of *sigB* inactivation on its expression confirmed that *bcp* is likely transcribed by σ^B^ associated with the RNA polymerase. Our results also indicate that the PerR- and OseR-independent induction of *bcp* by H_2_O_2_ or air is likely mediated by σ^B^. We have previously shown that the expression of *fdpF* and *revrbr1* is also strictly dependent on σ^B^ (18, 57), whereas *revrbr2* and *fdpA* are expressed under the dual control of σ^B^ and σ^A^ with the presence of two promoters (15). We showed that in the presence of H_2_O_2_, the expression of *revrbr1*, *revrbr2,* and *fdpF* was reduced in the *sigB::erm* mutant compared with the parental *perR*_WT_ strain (Fig. 8D). However, the impact of *sigB* inactivation was less important for the *revrbr2* gene likely due to the presence of the second σ^A^-dependent promoter. A downregulation of the expression of the *revrbr* and the *fdp* genes was also observed following air exposure in the *sigB::erm* mutant (Fig. S7E). Finally, we tested a possible involvement of σ^B^ in the control of the *rbr* operon. We observed a significant increase in the expression of *rbr*, *perR*, *sor,* and *CD0828* in the *sigB::erm* mutant compared with parental strain in anaerobiosis (Fig. 8E). However, this regulation disappeared in the presence of H_2_O_2_ or air except for the *rbr* gene (Fig. 8D; Fig. S7E). The negative control of the *rbr* operon by σ^B^ is indirect in agreement with the presence of a consensus recognized by σ^A^ upstream of the *rbr* operon TSS (56). As observed in the ∆*oseR* mutant (Fig. 8B), an additional level of control specific to the *rbr* gene probably exists (Fig. 8D).

### Regulation of *oseR* during stress exposure

**Fig 9:**
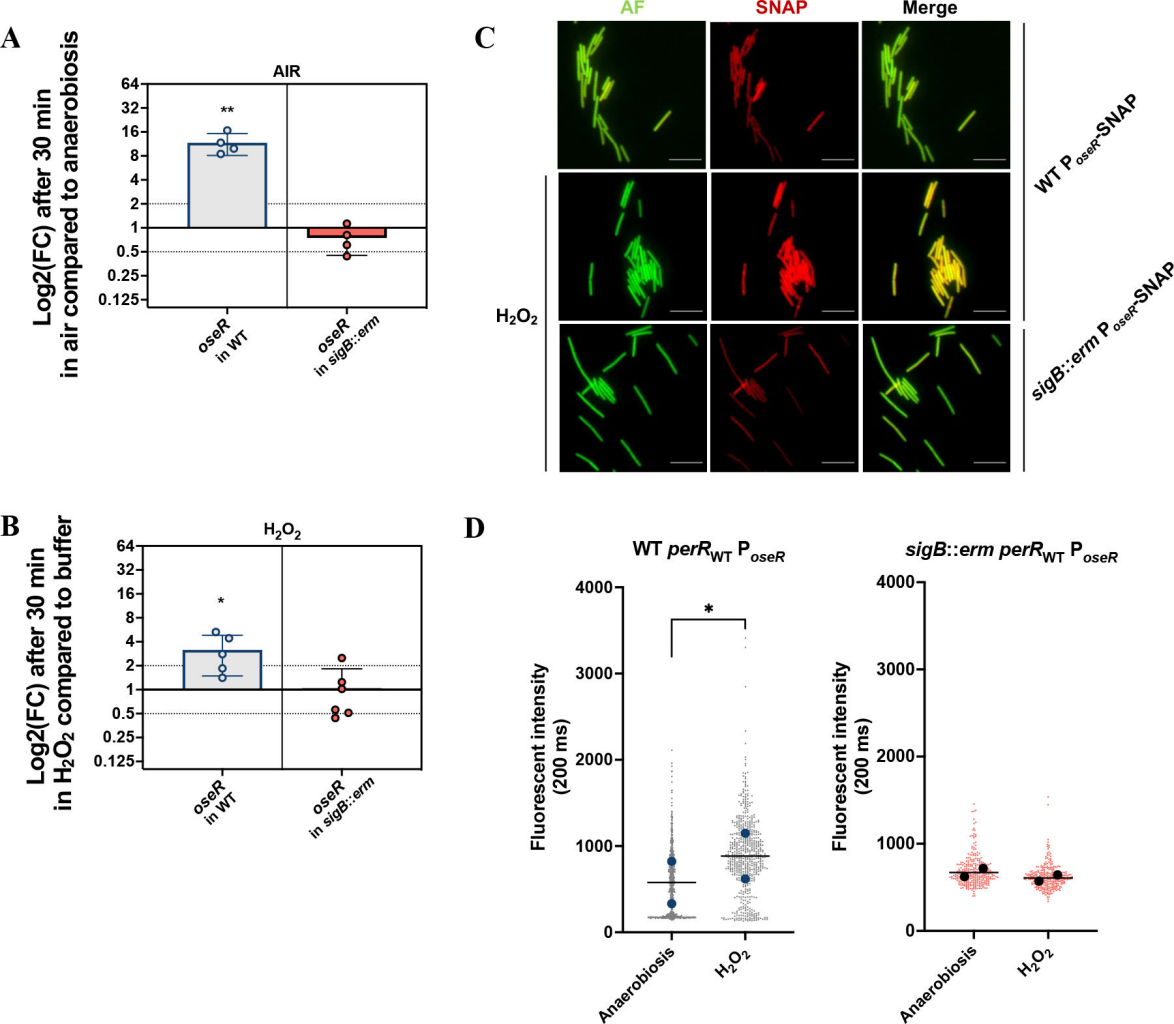
Regulation of *oseR* expression. (**A, B**) Expression of the *oseR* gene was evaluated by qRT-PCR in the 630∆*erm perR*_WT_ (WT) and the *perR*_WT_ *sigB::erm* strains. We compared the expression without or with 30 min of exposure to air (**A**) or 100 µM H_2_O_2_ (**B**). *pgi* or *gyrA* were used as reference genes. Experiments were performed on least four biological replicates. Mean and standard deviation (SD) are shown. One-sample *t*-tests were used with comparison of the fold change to 1. ns: not significant, *: *P*-value < 0.05 ; **: *P*-value < 0.01. (**C**) Pictures of *C. difficile* 630∆*erm perR*_WT_ and *perR*_WT_*sigB::erm* mutant strains carrying a transcriptional fusion P*_oseR_*-SNAP. Mid-exponential grown cells were resuspended in glycylglycine buffer and exposed or not for 30 min to 100 µM H_2_O_2_. Cells were observed at high magnification (60×). Images are overlays of bacterial autofluorescence (AF) (green) and SNAP (red). Scale bars represented 10 µm. (**D**) Superplots of P*_oseR_*-SNAP fluorescence intensity of individualized bacteria from acquired images of panel C. Individualized values and associated medians of P*_oseR_*-SNAP fluorescence of at least 400 cells from two independent experiments in the WT and *sigB::erm* strains were represented. One paired two-tailed *t*-test was performed. *: *P*-value < 0.05.

We have previously shown that the expression of the *oseR* gene increases upon long-term exposure to 1% O_2_ and is controlled by σ^B^ (15). We then wanted to test whether *oseR* expression is also induced following H_2_O_2_ or air exposure. We observed an increase in the expression of the *oseR* gene after 30 min of exposure to air or H_2_O_2_ (Fig. 9A and B). This induction was abolished in the *sigB::erm* strain in agreement with the presence of a consensus recognized by σ^B^ upstream of the TSS of the *oseR* gene (15). The induction of *oseR* expression by H_2_O_2_ or air is σ^B^-dependent as observed for *bcp*. To confirm and complete these results, we introduced a transcriptional P*_oseR_*-SNAP fusion in the *sigB::erm* mutant and the parental *perR*_WT_ strains. After 30 min exposure to H_2_O_2_, we showed a significant increase in the fluorescence intensity in the *perR*_WT_ strain that was absent in the *sigB::erm* mutant (Fig. 9C and D), in agreement with the RT-qPCR data.

## DISCUSSION

The anaerobic bacterium *C. difficile* has a multiplicity of ROS detoxification enzymes enabling its survival upon exposure to the ROS produced by immune cells or endogenously produced at the different O_2_ tensions encountered along the GIT. We demonstrated that Rbr, Sor, and CD0828 as well as Bcp are important for the survival of *C. difficile* in the presence of H_2_O_2_ or at high O_2_ tensions (4% or air). In vegetative cells, the two peroxidases, Rbr and Bcp, play a key role in H_2_O_2_ scavenging in agreement with the drastic reduction in survival observed in the corresponding mutants in the presence of H_2_O_2_. The biochemical characterization of the peroxidase activity of Bcp remains to be done, but we showed that the Rbr protein has a peroxidase activity *in vitro* as observed for the Rbrs of *Desulfovibrio vulgaris* and *Pyrococcus furiosus* (38, 40). In addition to these two peroxidases, FdpF and revRbr2 have dual O_2_- and H_2_O_2_-reductase activities *in vitro* (18, 25). We highlighted a minor physiological role of FdpF and revRbr2 in the *perR*_WT_ strain, which does not overproduce Rbr. It is worth noting that the *revrbr2* mutant, but not the *revrbr1* mutant, has a phenotype in the presence of H_2_O_2_, whereas only the *revrbr1* mutant is affected upon long-term exposure at 1% O_2_ (15) in accordance with the 3-fold higher H_2_O_2_-reductase activity of revRbr2 compared with revRbr1 (18). Despite their 96% protein identity, we observed a physiological specialization of these enzymes, with revRbr1 more associated with O_2_ and revRbr2 more associated with H_2_O_2_. In addition, a secreted glutamate dehydrogenase, GluD, also protects *C. difficile* from H_2_O_2_ by an unknown mechanism (58). This arsenal of defense systems probably favors *C. difficile* colonization and infection, protecting the bacterium from the H_2_O_2_ produced by the microbiota, the epithelial cells, and the host innate immune defense during inflammation but also endogenously by *C. difficile* itself.

We showed that Rbr has also some O_2_-reductase activity *in vitro*. In addition to the four different O_2_-reducing enzymes with different, yet overlapping, spectra of activity in a range of O_2_ tensions from 0.1% to 21% (15, 18), Rbr is a fifth potential O_2_-reductase. The Rbr role is marginal at 1% O_2_ but contributes to the protection of *C. difficile* at 4% O_2_ or in air. Under these conditions, H_2_O_2_ is produced. Rbr is probably mainly involved in the detoxification of endogenous H_2_O_2_ even if we cannot exclude a direct physiological role in O_2_-reduction at tensions ≥ 4%. Oxygenation of the intestinal epithelium increases upon antibiotic treatment and dysbiosis in conventional mice models of CDI (59, 60), so that during infection, *C. difficile* must be exposed to O_2_ tensions, triggering an endogenous production of H_2_O_2_ but maybe also O_2_^•−^. Studies have also shown that *C. difficile* is detected in the small intestine (61), and close to the mucus and epithelial cells following the disorganization of the intestinal barrier due to the action of toxins (62, 63).

SOR and SOD are O_2_^•−^ detoxifying enzymes. Although not restricted to anaerobes, SOR is predominantly found in anaerobic microorganisms, whereas the production by SOD of O_2_ from O_2_^•−^ is unsuitable for obligate anaerobes. The low SOD activity *in vitro* compared with the SOR activity has probably no physiological relevance. The growth defect of the *sor* mutant on menadione is similar to that of a *sor::erm* mutant of a RT027 strain with a disc diffusion assay but only after a short exposure to air (26). In our growth conditions in microplates, a very low O_2_ concentration is likely present, enough though to allow us to observe a growth defect associated with *sor* inactivation in the *perR*_WT_ strain. The ∆*sor* mutant is also affected upon exposure to O_2_ tensions ≥ 4%. In other obligate anaerobes, *D. vulgaris* and *Treponema denticola*, *sor* mutants also show an increased sensitivity to air (64, 65). In *B. thetaiotaomicron* (4, 66), O_2_^•−^ is produced when this anaerobe is exposed to O_2_. Surprisingly, we also detected a drop in survival of the ∆*sor* mutant following exposure to H_2_O_2_ in contrast with a prior analysis of an RT027 strain using a disc diffusion assay in rich medium (26). This difference is likely due to our CFU-based analyses of exponentially growing cells exposed to H_2_O_2_ being more sensitive than the disc diffusion assay. This phenotype is not directly due to a peroxidase activity of Sor, which it lacks, but rather to a more complex indirect impact. The excess of H_2_O_2_ may lead to Fenton chemistry and, ultimately to the Habber-Weiss chain reactions, which, in turn, lead to the formation of O_2_^•−^ (22, 67).

The Sor and Rbr enzymatic activities were carried out using the NROR and the Rd-D of Fdp from *E. coli* to transfer the electrons from NADH (18). CD0828, which contains a Rd domain and could be reduced by NADH, was able to transfer electrons to Rbr and Sor, but at a very long timescale, which likely indicates that CD0828 is not a physiological partner for these enzymes. Nevertheless, except for the *perR*_WT_ strain at 4% O_2_, we observed similar phenotypes for the ∆*CD0828* and ∆*rbr* mutants after exposure to H_2_O_2_, 4% O_2_, or air, whereas this is not the case for the ∆*sor* mutant. Neither the amino acid sequence nor the predicted structure of CD0828 corresponds to a canonical NROR protein. In addition, when interrogating Foldseek server (37) for proteins with structures or predicted models similar to CD0828, we identified similarities with an archaeal family of glutamate synthase from *M. jannaschii*. CD0828 could either correspond to a new protein family featuring both an Rd domain and a new type of Rd-reductase domain, or the fusion of a Rd with a glutamate synthase-like domain with unknown function. In *C. difficile*, the glutamate dehydrogenase GluD is involved in ROS protection (58). Its product, α-ketoglutarate, is important for managing oxidative stress in both prokaryotes and eukaryotes (68). We cannot exclude that CD0828 can interfere with the metabolism of glutamate and can be involved in ROS protection independently of Rbr or Sor activity. As it happens in other organisms, partners of Rbr and Sor remain to be identified in *C. difficile*. Similarly, Bcp partners remain to be identified. Trx systems are involved in the reduction of the enzymes of the Bcp/PrxQ family (69). Among the three Trx systems of *C. difficile*, it would be interesting to determine which one is involved in Bcp regeneration. It is likely one or the two systems dedicated to stress response (70).

Our phenotype analysis was done in *perR*_WT_ and *perR*_mut_ backgrounds. The *perR*_mut_ strain is more tolerant to H_2_O_2_ and air exposure due to the overexpression of the *rbr* operon caused by the point mutation in the helix-turn-helix motif of the PerR repressor, preventing its binding to DNA (27). PerR regulates neither the *bcp* gene nor the *revrbr1*, *revrbr2,* or *fdpF* genes (15) in agreement with the absence upstream of these genes of the putative PerR binding site identified in the *rbr* promoter region (32). The PerR regulon is rather restricted in *C. difficile* compared with *C. acetobutylicum* (71). As observed in other Bacillota, the PerR repressor seems to be inactivated following an H_2_O_2_ exposure probably through its oxidation state (27). The regulation of the *rbr* operon also involves σ^B^ and OseR. Its transcription from a promoter recognized by σ^A^, indicates an indirect effect of σ^B^. This control is likely mediated through the OseR regulator, as *oseR* is transcribed from a σ^B^-dependent promoter (15). In the *sigB* mutant, the drastic reduction in the expression of *oseR* leads to a derepression of the *rbr* operon in anaerobiosis as observed in the ∆*oseR* mutant. For the *revrbr* and *fdp* genes, the regulation pattern is more complex with a double effect of σ^B^, direct at the level of transcription initiation, and indirect through the control of *oseR* transcription (15, 18, 31). In anaerobiosis, the positive direct impact of σ^B^ on the initiation of transcription of the *fdp* and *revrbr* genes is epistatic over the negative OseR-dependent control, explaining the different pattern of expression observed for the σ^A^-dependent *rbr* operon. By contrast, the control of *bcp* expression in response to oxidative stress (H_2_O_2_, air) is OseR-independent but dependent on σ^B^, which directly transcribes *bcp* (56). The involvement of σ^B^ in the *bcp* induction by H_2_O_2_ is reminiscent of a previous work proposing that H_2_O_2_ could be a signal triggering σ^B^ activation (72).

**Fig 10:**
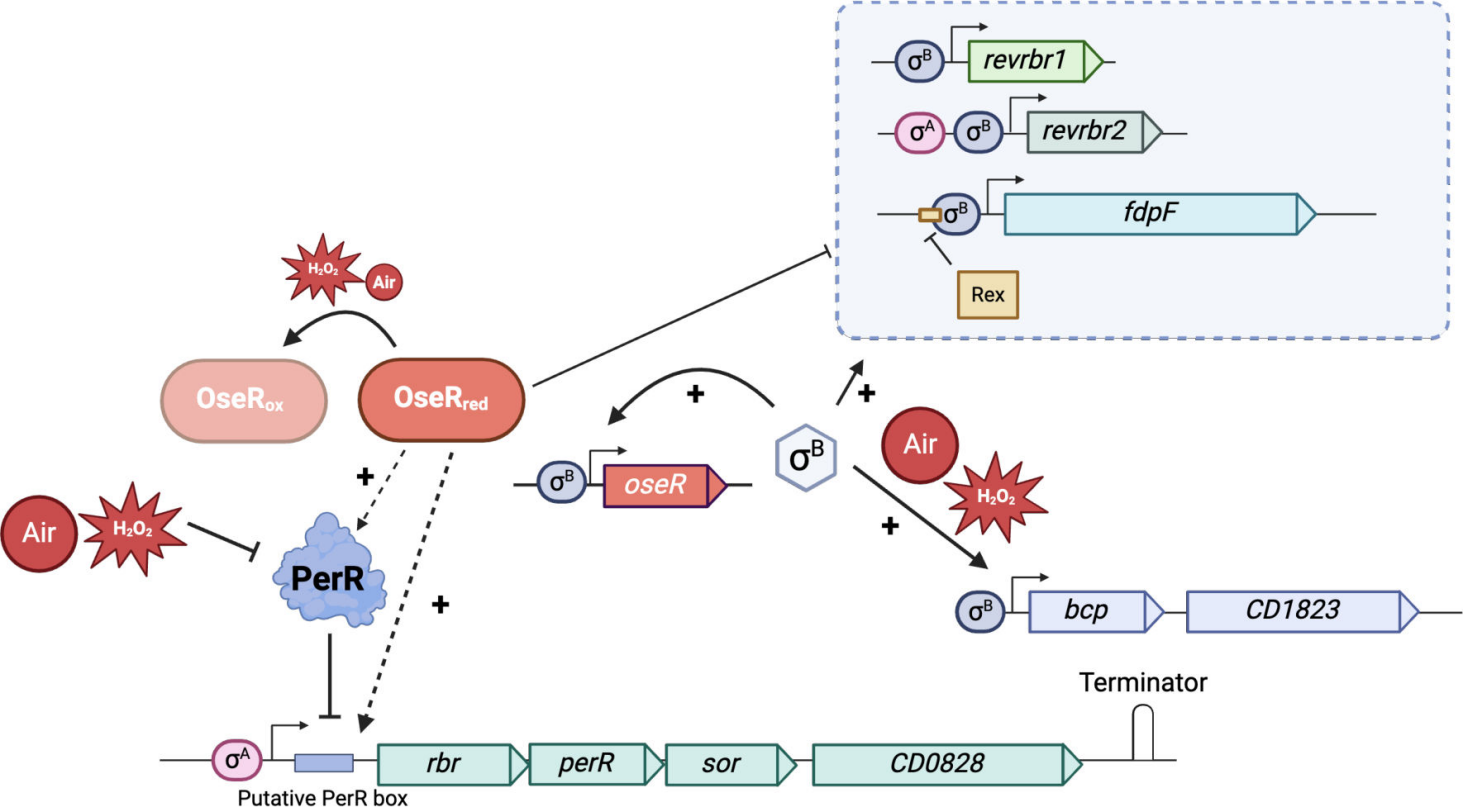
Model of control of genes encoding ROS-detoxification enzymes. Model of the transcriptional regulation of the genes encoding the ROS-reductases. The genes *bcp, fdpF*, *revrbr1, revrbr2,* and *oseR* are expressed under the control of σ^B^, whereas the *rbr* operon and *revrbr2* are expressed under the control of σ^A^-dependent promoters. H_2_O_2_ and air induce the expression of these genes. Depending on the genes, this induction is mediated by PerR, OseR, and/or σ^B^.

OseR represses the expression of the *rbr* operon in anaerobiosis. Its induction upon exposure to H_2_O_2_ or air is mediated by PerR (27) but maybe also by OseR. Indeed, in the ∆*oseR* mutant, the derepression of not only the *rbr* operon but also the *revrbr* and *fdpF* genes is abolished in the presence of H_2_O_2_ or air, although we have previously shown that the *revrbr* and *fdp* genes are also induced upon long-term exposure to 1% O_2_ through OseR. OseR might detect both O_2_ and H_2_O_2_ (Fig. 10). The mechanism of sensing oxidative stress by OseR may involve the oxidation of a redox-sensing cysteine at a CXXV motif (15). This is reminiscent of the redox sensing switch at a CXXC motif of Spx in several Bacillota (73). It is also well established that the Spx-type regulators contribute to the H_2_O_2_ response in other Bacillota (73). The degree and rate of OseR cysteine oxidation might differ, depending on the signaling molecule (O_2_ or ROS) and the intensity of oxidative stress. Then, OseR could recognize a motif upstream of the *rbr* operon and control this operon independently of PerR (Fig. 10). Alternatively, OseR might interfere with the PerR regulation by modulating the internal concentration of its effector, H_2_O_2_ or the conformation of PerR. Finally, although the induction of *perR*, *sor,* and *CD0828* by H_2_O_2_ is lost in an ∆*oseR* mutant, a residual induction is specifically detected for *rbr,* suggesting an additional mechanism of control such as a non-coding RNA (74).

In conclusion, the regulation of the genes encoding the ROS-reductases of *C. difficile* is complex (Fig. 10). As previously described (15, 18), the OseR regulator and the sigma factor, σ^B^, are involved in the control of the *revrbr* and *fdp* genes, but H_2_O_2_ is a second signal triggering the expression of the *revrbr* genes and *fdpF* in addition to low O_2_ tension. We observed a different regulation for the *bcp* gene and the *rbr* operon. Although the *bcp* gene is mainly expressed under the direct control of σ^B^, a more complex regulatory network controls the expression of the *rbr* operon involving the PerR repressor sensing H_2_O_2_ but also a cascade of regulation involving σ^B^ and OseR. A non-coding RNA whose expression would be induced or repressed upon H_2_O_2_ exposure could specifically target the *rbr* gene. In addition, another level of control exists with the σ^B^-dependent induction of *oseR* expression in the presence of H_2_O_2_, air or long-term exposure to 1% O_2_ (15). The interplay of these different regulations could fine tune the expression of the genes encoding this armada of ROS-reducing enzymes adapting the response to different sources and intensities of oxidative stress. This complex and differential regulation might favor *C. difficile* colonization under conditions of higher oxygenation in a dysbiotic colon and oxidative burst mediated by the neutrophils recruited to the site of infection.

## MATERIALS AND METHODS

### Strains and growth conditions

The *C. difficile* and *E. coli* strains and the plasmids used in this study are listed in Table S1. *E. coli* strains were grown aerobically in LB broth (tryptone 10 g L^−1^, yeast extract 5 g L^−1^, NaCl 5 g L^−1^). When indicated, ampicillin (Amp, 100 µg mL^−1^) and chloramphenicol (Cm, 15 µg mL^−1^) were added to the medium. *C. difficile* strains were grown anaerobically (5% H_2_, 5% CO_2_, 90 N_2_) in TY (Bacto tryptone 30 g L^−1^, yeast extract 20 g L^−1^, pH 7.4) or in brain heart infusion (BHI; Difco). For solid media, agar was added to a final concentration of 17 g L^−1^. When necessary, thiamphenicol (Tm, 15 µg mL^−1^), cefoxitin (Cfx, 25 µg mL^−1^), and cycloserine at 250 µg mL^−1^ were added to *C. difficile* cultures. When mentioned, taurocholate (Tau) was added at 0.05%(wt/vol) for some phenotypic tests to favor spore germination.

### Construction of mutant strains

We deleted the *sor* and *bcp* genes by the allelic chromosomic exchange (ACE) method using the pMSR vector (75). Plasmids with the flanking regions of the *sor* or the *bcp* genes were constructed using Gibson Assembly (New England Biolabs). Upstream (part I) and downstream (part II) regions were amplified by PCR using primers IMV1311/1312 and IMV1313/1330 for *sor* and LC4/LC5 and LC6/LC7 for *bcp*. The pMSR vector was linearized by inverse PCR using primers IMV910/911 and then treated with the DpnI enzyme to eliminate the original plasmid. The absence of mutation in the plasmids was checked by sequencing. These plasmids pDIA7123 (ACE *sor perR*_mut_), pDIA7139 (ACE *sor perR*_WT_), and pDIA7293 (ACE *bcp*), as well as pDIA6887 (ACE *revrbr1*), pDIA6888 (ACE *revrbr2*), and pDIA6893 (ACE *fdpF*) (Table S1), were introduced in the HB101/RP4 *E. coli*. They were then transferred by conjugation into *C. difficile* strains. Transconjugants were selected on BHI plates supplemented with Tm and *C. difficile* selective supplement (SR0096, Oxoid). As plasmids derived from pMSR are unstable, clones with bigger colonies are those having integrated the plasmid in the chromosome by a single recombination event. These clones were then restreaked on BHI plates containing aTc at 200 ng mL^−1^, allowing the expression of the *CD2517.1* toxin gene cloned under the control of the P_tet_ promoter and selecting the double crossover events (75). Verification of the deletion was performed by PCR with the primers IMV1314/611 for the ∆*sor* mutant and LC8/LC9 for the ∆*bcp* mutant. Verification of the deletion of *revrbr1*, *revrbr2,* and *fdpF* was performed as previously described (18). Steps were repeated in each different mutant to generate *C. difficile* multi mutants (Table S1). Primers are listed in Table S2.

We also obtained a pMSR ACE *rbr* plasmid (pDIA7120) carrying a fragment upstream of *rbr* (IMV1307/1308) and another downstream of *rbr* (IMV1309/1310). However, we failed to obtain deletion of the *rbr* gene using the ACE method. Hence, we used an alternative strategy based on the use of an endogenous CRISPR system (76). First, a partial “CRISPR miniarray” sequence was prepared by hybridization of long primers LC2 and LC3 after denaturation at 100°C and subsequent rehybridization. Insertion of the “CRISPR miniarray” was performed by “Gibson assembly” in the pDIA6555 plasmid (76) amplified by inverse PCR and treated with the DpnI enzyme. The plasmid with the “CRISPR miniarray” targeting *rbr* was then digested by SmaI to add the flanking regions of *rbr* obtained by PCR using primers IMV1338/1339 and the plasmid pDIA7120 (pMSR-ACE *rbr*). The PCR product was digested with StuI before ligation. The pDIA7147 plasmid (CRISPR *rbr*) was purified, and the absence of mutation was checked by sequencing. This plasmid was then introduced in the HB101 *E. coli* strain and transferred by conjugation in the 630∆*erm perR_mut_* and 630∆*erm perR*_WT_ strains. Clones were then restreaked on BHI plates containing 500 ng/mL of aTc. The addition of aTc allows the induction of expression of the crRNA specific to the *rbr* gene and thus to counter-select clones where there were no allelic exchanges. Verification of the deletion was performed by PCR with the primers IMV1318 and IMV828.

### Complementation of the deletion mutant strains

Plasmids derived from the pMTL84121 vector were constructed for complementation. We amplified *rbr* using primers LC12 and LC13 and cloned by Gibson assembly the gene with its promoter region in the pMTL84121 vector linearized by inverse PCR using primers CM13 and IMV993 to give pDIA7121. The *sor* and the *CD0828* genes were amplified using primers IMV1334/1335 and IMV1523/1524, respectively. The promoter of the *rbr* operon was amplified with primers IMV1318/1320. By a Gibson Assembly strategy, we then cloned the P*_rbr_* promoter and the *sor* or *CD0828* gene in the pMTL84121 vector linearized by inverse PCR. The *bcp* gene and its promoter region were amplified by PCR using LC10/LC11 and cloned into pMTL84121. Plasmids pDIA7121 (pMTL84121-P*_rbr_-rbr*), pDIA7125 (pMTL84121-P*_rbr_-sor*), pDIA7257 (pMTL84121-P*_rbr_-CD0828*), and pDIA7291 (pMTL84121-P*_bcp_-bcp*) were sequenced to check the absence of mutations. These plasmids were then transferred by conjugation into the corresponding mutants. Despite several attempts, we failed to transfer the plasmid pMTL84121-P*_rbr_-rbr* in the *perR*_mut_ strain. The level of production of Rbr in this strain might be toxic. Therefore, we expressed *rbr* under the control of the inducible P_tet_ promoter, by amplifying *rbr* by PCR using IMV1653 and IMV1654. The PCR product was then digested by StuI and BamHI and cloned into pDIA6103 (74) to produce pDIA7327 (Table S2). This plasmid was then transferred by conjugation in the 630∆*erm perR*_mut_ ∆*rbr* strain.

### Protein production, purification, and quaternary structure determination

The coding regions of *rbr*, *sor,* and *CD0828* were amplified by PCR using genomic DNA or an optimized sequence for *E. coli* (*CD0828*) and primer pairs IMV1369/IMV1371, IMV1372/IMV1374, or AL01/AL02, respectively. The PCR products were cloned into pET22 (Novagen), yielding pDIA7151, pDIA7153, or pDIA7276, respectively. After verification by sequencing, the plasmids were introduced in *E. coli* BLI5. Strains overproducing Rbr and Sor were grown aerobically at 37°C at 150 rpm in M9 minimal medium supplemented with 20 mM glucose, 0.1 mM FeSO_4_, and Amp. When the OD_600_ reached 0.4, 0.1 mM FeSO_4_ was added, and gene expression was induced by the addition of 0.1 or 1 mM isopropyl-β-D-thiogalactopyranoside (IPTG), respectively. After 16 h of growth at 30°C, the cells were harvested by centrifugation, resuspended in a buffer containing 50 mM Tris-HCl (pH 7.5), and stored at −20°C. Strain overproducing CD0828 was also grown aerobically at 37°C in M9 minimal medium, supplemented with Amp. When OD_600_ reached 0.6, 0.1 mM IPTG and 0.1 mM FeSO_4_ were added, and the temperature was lowered to 25°C. After 16–18 h growth, the cells were harvested by centrifugation, resuspended in buffer containing 50 mM Tris-HCl (pH 7.5), and stored at −20°C. Cells were disrupted by at least three cycles in a French press apparatus at 16,000 lb/in² (Thermo) in the presence of DNase (Applichem). For CD0828, cells were disrupted by three cycles in Emulsiflex apparatus at 10,000 psi, in the presence of DNase. The crude extracts containing Rbr, Sor, or CD0828 were cleared by low-speed centrifugation at 25,000 × *g* for 25 min and then at 138,000 × *g* for 90 min at 4°C to remove cell debris and the membrane fraction, respectively. For Rbr, the soluble extract was dialyzed overnight at 4°C against buffer A (20 mM Tris-HCl [pH 7.5], 18% glycerol) and subsequently loaded onto a Q-Sepharose Flow column (65 mL; GE Healthcare) previously equilibrated with buffer A. For CD0828, the soluble extract was directly loaded onto a Q-Sepharose Flow column, using the same buffer. For Sor, the buffer was similar only with 5% glycerol instead of 18%. Proteins were eluted with a linear gradient from buffer A to buffer B (buffer A containing 500 mM NaCl). The eluted fractions were monitored throughout the purification process by SDS-PAGE and UV-visible spectroscopy. Fractions containing the desired protein were pooled and concentrated. For Rbr and Sor, the concentrated fraction was then loaded onto a size exclusion Superdex S75 column (330 mL; GE Healthcare) equilibrated with buffer A containing 150 mM NaCl, whereas for CD0828, the concentrated fraction was loaded onto a Superdex S200 column (330 mL; GE Healthcare). The fractions containing the desired proteins (excluding the high molecular aggregates) were pooled and concentrated. Fractions containing purified proteins were verified by SDS-PAGE (Fig. S2A through C).

The quaternary structures of the proteins were determined by size exclusion chromatography. Proteins were loaded onto a 25 mL Superdex S200 10/300 Gl column (GE Healthcare) previously equilibrated with buffer A containing 150 mM NaCl (for the Sor, the buffer did not contain glycerol). A mixture containing tyroglobulin (669 kDa), apoferritin (443 kDa), β-amylase (200 kDa), alcohol dehydrogenase (150 kDa), albumin (66 kDa), carbonic anhydrase (29 kDa), and dextran blue (2,000 kDa) as a void volume marker was used as the standard (Fig. S2D and E). For CD0828, the standard mixture contained ovalbumin (44 kDa), conalbumin (75 kDa), aldolase (158 kDa), ferritin (440 kDa), thyroglobulin (669 kDa), and blue dextran (2,000 kDa) as a void volume marker (Fig. S2F).

### Protein and metal quantification

Purified protein samples were quantified using a BCA kit (Thermo) and bovine serum albumin as the standard. The iron content was determined by the phenanthroline colorimetric method. Protein samples were incubated for 15 min with 8 M HCl and for a further 30 min with 8% trichloroacetic acid at room temperature, centrifuged at 8,000 × *g* for 5 min. Samples were then incubated with 10% hydroxylamine and 0.3% 1–10-phenanthroline. The absorbance spectrum was measured, and the iron content was quantified by using ε_510_ = 11.2 mM^−1^ cm^−1^. The iron content was further confirmed using inductively coupled plasma emission.

### Spectroscopic methods and redox titration

UV-visible spectra were obtained in a Perkin-Elmer Lambda 35 spectrophotometer. A fully oxidized Sor sample was prepared aerobically by incubation with an excess of potassium hexachloroiridate.

The reduction potential of the center I of Sor was determined by an anaerobic redox titration monitored by visible absorption spectroscopy in a Shimadzu UV-1603 spectrophotometer. Protein samples at a 30 µM final concentration in buffer C (50 mM Tris-HCl [pH 7.5] and 18% glycerol) were titrated inside an anaerobic chamber (Coy Lab Products) by the stepwise addition of a buffered sodium dithionite solution in the presence of a mixture of redox mediators (0.5 µM each): *N*,*N*-dimethyl-*p*-phenylenediamine (*E*′° = +340 mV), dichlorophenolindophenol (*E*′° = +217 mV), 1,2 naphtoquinone-4-sulfonic acid (*E*′° = +215 mV), 1,2 naphtoquinone (*E*′° = +180 mV), trimethylhidroquinone (*E*′° = +115 mV), phenazine methosulfate (*E*′° = +80 mV), 1,4 naphtoquinone (*E*′° = +60 mV), phenazine ethosulfate (*E*′° = +55 mV), 5-hydroxy- 1,4-naphtoquinone (*E*′° = +30 mV), duroquinone (*E*′° = +5 mV), menadione (*E*′° = +0 mV), plumbagin (*E*′° = –40 mV), resorufin (*E*′° = –51 mV), indigo trisulfonate (*E*′° = –70 mV), indigo disulfonate (*E*′° = –110 mV), phenazine (*E*′° = –125 mV), 2,5-hydroxy-*p*-benzoquinone (*E*′° = –130 mV), 2-hydroxy-1,4-naphtoquinone (*E*′° = –152 mV), and phenosafranine (*E*′° = –255 mV). A combined Pt electrode (Ag/AgCl in 3.5 M KCl, as a reference) was used and calibrated at 23°C against a saturated quinhydrone solution (pH 7). The reduction potential of the neelaredoxin site (Center II) could not be determined, since in those conditions, it remained fully reduced and could not be oxidized. The absorption values obtained at 490 nm (typical of the oxidized Dx-like Center I site) were normalized in relation to the full oxidized protein, and a theoretical curve of the oxidized population was fitted using a SciLab routine and applying a single Nernst equation for a one-electron transfer process. The populations were calculated as follows:


$$E=E^{0}+RT\;ln\;\left(Q\right),$$

where Q is calculated as follows:


$$Q=\frac{\left[Rd\right]Ox}{\left[Rd\right]Red}$$

Therefore,


$$\begin{array}{rl} & [Rd]\text{ Red }=\frac{10^{\frac{E0-E}{Y}}}{1+10^{\frac{E0-E}{Y}}}\\ & [Rd]Ox=1-[Rd]\text{ Red } \end{array}$$

Where$\;\;Y=2.303\;\frac{RT}{nF}$

where *R* = 8.314 J K^−1^ mol^−1^, *T* = 298.15 K, *n* = 1 electron, and *F* = 96,485 C mol^−1^.

The theoretical model and curve adjustment were performed using Scilab 6.0.2. The *E*_0_ for the Rd center of the Sor was determined from the fit.

### Spectrophotometric measurement of the H_2_O_2_- and O_2_-reductase activities

Because the physiological electron donor to the *C. difficile* Rbr and Sor is unknown, we used an artificial electron-donating system: a mixture of the Rd-D of the *E. coli* Fdp and of the Fdp reductase (NROR, gene *norW*, *E. coli* strain K-12). These *E. coli* proteins were purified as previously described (50). The enzymatic activity for H_2_O_2_ was determined by UV-visible spectroscopy, inside an anaerobic chamber. The assays were performed in 50 mM Tris (pH 7.5), 18% glycerol. The reaction was monitored at 340 nm, determining the NADH consumption (ε_340_ = 6,220 mM^−1^ cm^−1^). A mixture of buffer containing NADH (200 µM), NROR (0.4 µM), and Rd-D (2 µM) was used. Different amounts of NROR and Rd-D were tested and optimized in combination with each enzyme to ensure that the reaction rates were maximized. Concentration of Rbr was 1 µM. The reaction was initiated by the addition of H_2_O_2_ at different concentrations (10–200 µM) to evaluate the dependence of the rates on the amount of substrate. The calculated rates (s^−1^) presented in Table 1 were calculated by subtracting the experimental slope (µM.s^−1^) before and after the addition of H_2_O_2_, divided by the protein concentration (µM).

The O_2_-reductase activity of the Rbr was measured indirectly by UV-visible spectroscopy, at 25°C in an air-equilibrated buffer (about 260 µM O_2_). The reaction was monitored at 340 nm, determining the NADH concentration (ε_340_ = 6,220 mM^−1^ cm^−1^). A mixture of buffer (50 mM Tris [pH 7.5], 18% glycerol) containing NADH (200 µM), NROR (0.4 µM), and Rd-D (2 µM) was used. The reaction was initiated by the addition of Rbr (2 µM) after stabilization of the NROR indirect activity to distinguish both activities. Assays were performed in the presence of catalase (640 nM). The calculated rates (s^–1^) presented in Table 1 were calculated by subtracting the experimental slope (µM s^−1^) before and after the addition of the Rbr divided by the protein concentration (µM).

### Measurement of superoxide reductase and superoxide dismutase activities

The O_2_^•−^-reductase activity of the Sor enzyme was measured indirectly by UV-visible spectroscopy, at 25°C in air. The reaction was monitored at 490 nm, determining Rd-D oxidation (ε_490_ = 8,371 mM^−1^ cm^−1^). A mixture of buffer (50 mM Tris, pH 7.5) containing NADH (40 µM), NROR (1 µM), and Rd-D (30 µM) was used; 1.5 mM of xanthine and 0.046 units mL^−1^ of xanthine oxidase were added to generate a continuous flux of O2^•−^ of around 7.7 µM min^−1^. This addition leads to a slow reoxidation of the Rd-D. After a few seconds, the addition of Sor (0.006–0.06 µM) increased the Rd-D reoxidation. Assays were performed in the presence of catalase (640 nM). The oxidation rates of Rd-D were measured before and after the addition of the Sor for each condition. The rates in the presence of various Sor concentrations were used to calculate the *k*_app_ value. In brief, the *k*_app_, the slope of the linear regression for the representation of *v_0_* (μM min^−1^), is plotted against Sor concentrations used in each assay (μM) (77). As the concentration of both Rd and O_2_^•−^ used in each assay was significantly larger than Sor, we considered them constant, then


$$v_{0}=k\;[Rd]\;[O_{2}^{∙-}]\;[Sor]$$

becomes


$$\begin{array}{rl} & v_{0}=k_{app}\;[Sor]\\ & where\;k_{app}\;is\\ & k_{app}=k\;[Rd]\;[O_{2}^{∙-}] \end{array}$$

The O_2_^•−^-dismutase activity of the Sor enzyme was measured indirectly by UV-visible spectroscopy at 25°C in air. The reaction was monitored at 550 nm, determining the cytochrome *c* reduction rate (ε_550_ = 21 mM^−1^ cm^−1^). A mixture of buffer (50 mM Tris, pH 7.5) containing 10 µM of horse heart cytochrome *c* was used; 1.5 mM of xanthine and 0.046 units mL^−1^ of xanthine oxidase were added to generate a continuous flux of O_2_^•−^ of around 7.7 µM min^−1^. This addition leads to a slow reduction in cytochrome *c*. After a few seconds, the addition of Sor (0.09–0.6 µM) leads to a decrease in cytochrome *c* reduction rate. Assays were performed in the presence of catalase (640 nM) to avoid the side effects of the presence of H_2_O_2_. The reduction rates of cytochrome *c* were compared with and without the addition of the Sor for each condition. Similarly to the SOR assays, the *k*_app_ is the slope of the linear regression for the representation of *v_0_* (μM min^−1^) plotted against Sor concentrations used in each assay (μM).

### CD0828 optimal electron donor and electron transfer to Rbr and Sor

To evaluate the ability of CD0828 to act as an electron donor for the Rbr and Sor, its optimal electron donor was determined. CD0828 reduction by NADH and NAD(P)H was first evaluated, anaerobically, by the stepwise addition of 0.25 eq. (up to two eq.) of these reagents, to a reaction mixture containing 40 µM of CD0828 in 100 mM MOPS (pH 7.5) and 150 mM NaCl. These assays were monitored by UV-visible spectroscopy (UV-1800 Shimadzu). Then, the CD0828 reduction rates with either NADH or NADPH were measured by UV-visible spectroscopy at 450 nm over time under anaerobic conditions, in 100 mM MOPS (pH 7.5) and 150 mM NaCl. In brief, to a reaction mixture containing 20 µM of CD0828, we added 200 µM of NADH in the presence or absence of 40 µM of FMN. Similar assays were performed using NADPH as an electron donor. After determining the optimal electron donor for CD0828, Sor’s reduction using this protein was measured anaerobically by UV-visible spectroscopy at 513 nm. The assays were performed in 100 mM MOPS, pH 7.5, and 150 mM NaCl. A mixture of 10 µM CD0828, 30 µM Sor, and 20 µM FMN was used. Reaction was initiated by the the addition of 200 µM NADH. The Rbr’s reduction using CD0828 was performed in the same conditions as the ones described for Sor but using 40 µM of Rbr.

### Survival assays in presence of H_2_O_2_ or various O_2_ tensions

*C. difficile* strain was cultured in TY supplemented with 0.05% Tau overnight. Then, a fresh culture of TY Tau was inoculated at 1:50. After 3 h of growth, we prepared an inoculum at an OD_600_ of 0.5. For each strain, serial dilutions by 10 up to 10^−5^ were prepared; 5 µL of each dilution and the non-diluted inoculum were plated on calibrated square plates containing 28 mL of TY Tau agar to avoid the presence of spores. A control plate was kept at 37°C in anaerobiosis for 24 h. The other plates were incubated at 37°C either in hypoxia (5% CO_2_, × % O_2_, 95× % N_2_; BugBox M from Baker Ruskinn) or in air (21% O_2_) for various durations: 24 h at 1% O_2_, 8 or 16 h in the presence of 4% O_2_ and 1 or 2 h in air. Plates exposed to those conditions were subsequently incubated again for 24 h at 37°C in anaerobiosis. CFUs were numerated after incubations. Experiments were performed in five biological replicates.

*C. difficile* strain was cultured in BHI overnight. Then, a fresh culture was inoculated in BHI at 1:50. After 5 h of growth, we prepared an inoculum at an OD_600_ of 0.5 resuspended in 1 mL of glycylglycine buffer (50 mM glycylglycine and 0.2% glucose, pH 8) after washing; 100 or 400 µM of H_2_O_2_ (Honeywell) was added to the sample and incubated during 30 min. After treatment, serial dilutions were made down to a 10^−5^ dilution in BHI medium; 10 µL of all the dilutions and the non-diluted inoculum were spread for numeration. Cultures without stress were spread as controls. The results presented are the percentage of survival between the CFUs counted without stress compared with the CFUs counted after 30 min of treatment.

### Growth in presence of menadione

To assess the impact of menadione on the growth of *C. difficile*, we performed 24 h growth kinetics using a culture diluted to OD_600_ 0.05 directly from an overnight culture in TY. Menadione was added at a final concentration of 5 µM to 1 mL of culture; 200 µL per well was then dispersed in a filmed 96-well plate. OD_600_ measurements were taken every 30 min with 30 s agitation at 300 rpm before each reading with a microplate reader at 37°C (Spectro star, Nano BMG Labtech).

### H_2_O_2_ colorimetric quantification assay

The Pierce Quantitative Peroxide Assay Kit (Thermo Scientific, Germany) was used according to the manufacturer’s instructions. *C. difficile* strains grown in BHI medium overnight were diluted 1:50 in 1 mL fresh BHI. These samples were dispatched in a 6-well plate and incubated in anaerobiosis in glycylglycine buffer, in air for 1 h or in 1% and 4% O_2_ during 24 h (BugBox M from Baker Ruskinn). Then, the supernatant was collected by centrifugation at 10 000 × *g* for 1 min. A calibration range of 9 known H_2_O_2_ concentrations from 0 to 125 µM was carried out on the same plate. OD_560_ was measured using a GloMax Explorer plate-reader (Promega). At least three independent cultures were used per group.

### RNA extraction and RT-qPCR

Samples resuspended in glycylglycine buffer were exposed for 30 min to H_2_O_2_ (100 µM for *perR*_WT_ background and 250 µM for *perR*_mut_ background) with the exception of the *sigB::erm* mutant of the *perR*_WT_ strain exposed during 15 min. Culture in TY Tau in 6-well plates was exposed to air for 30 min. After H_2_O_2_ or air exposure, the pellets were conserved at −80°C. For RNA extraction, the pellets were resuspended in the RNApro solution (MP Biomedicals), and RNA was extracted using the Direct-Zol RNA Miniprep kit (Zymo Research, Ivrine, USA). cDNAs synthesis and real-time quantitative PCR were performed as previously described (74, 78). In each sample, the quantity of cDNAs of a gene was normalized to the quantity of cDNAs of the *gyrA* or *pgi* genes. The relative change in gene expression was recorded as the ratio of normalized target concentrations (the threshold cycle ∆∆Ct method) (79). Primers are listed in Table S2. Experiments were performed in at least four biological replicates.

### Transcriptional SNAP fusions and microscopy analysis

To construct a transcriptional fusion of *oseR* with the SNAP reporter gene, we cloned a PCR fragment corresponding to the promoter regions of *oseR* using the AL12/AL13 primers (*oseR*) into the pFT47 vector (80). We monitored the expression of the *oseR* promoter following stress exposure (30 min H_2_O_2_). Then, we performed SNAP labeling and fluorescence microscopy as previously described (18). The images were taken with exposure times of 200 ms or 300 ms for SNAP (TRITC) canal and 800 ms for *C. difficile* autofluorescence in FITC canal. The cells were observed on a Nikon Eclipse TI-E microscope 60× objective and captured with a CoolSNAP HQ2 Camera. For quantification of the SNAP-TMR Star signal, resulting from transcriptional fusions, the mean fluorescence intensity of each bacterium was determined using Image J. At least 400 bacteria from two biological replicates were analyzed per condition.

### Statistical analysis and data presentation

For survival assays after stress, ordinary one-way ANOVA was performed followed by Dunett’s multiple comparison tests with the 630∆*erm* strain *perR*_mut_ or *perR*_WT_ with a confidence interval of 95%. For survival assays, a comparison between single ∆*bcp* and double deletion mutants ∆*rbr* ∆*bcp*, Mann-Whitney comparison test was performed. For qPCRs, one-sample *t*-tests were realized with a comparison of the fold-change to 1. For microscopy analysis, the means of the fluorescence data for each biological replicate were used to overlay on the dot plot, and statistical significance was calculated using a paired two-tailed *t*-test. All data are presented as the mean value ± standard deviation (SD). Bar plots, curves, Superplots, and statistical analysis were performed using GraphPad Prism (10.0.1) (San Diego, CA, USA). Figure 9 was produced with BioRender.com. Asterisks indicate *P*-values as follows: *, < 0.05; **, <0.01; ***, <0.001; and ****, <0.0001.

## SUPPLEMENTAL MATERIAL

Supplemental figures mbio.03753-24-s0001.pdf Figures S1 to S7.

Supplemental tables mbio.03753-24-s0002.docx Tables S1 and S2.

ASM does not own the copyrights to Supplemental Material that may be linked to, or accessed through, an article. The authors have granted ASM a non-exclusive, world-wide license to publish the Supplemental Material files. Please contact the corresponding author directly for reuse.
