# Supplementary material for: Defense arsenal of the strict anaerobe Clostridioides difficile against reactive oxygen species encountered during its infection cycle
Source: mBio. 2025 Mar 20;16(4):e03753-24. doi: 10.1128/mbio.03753-24 (PMC11980386; doi:10.1128/mbio.03753-24)
Supplement: Supplemental figures — Figures S1 to S7. [file mbio.03753-24-s0001.pdf]

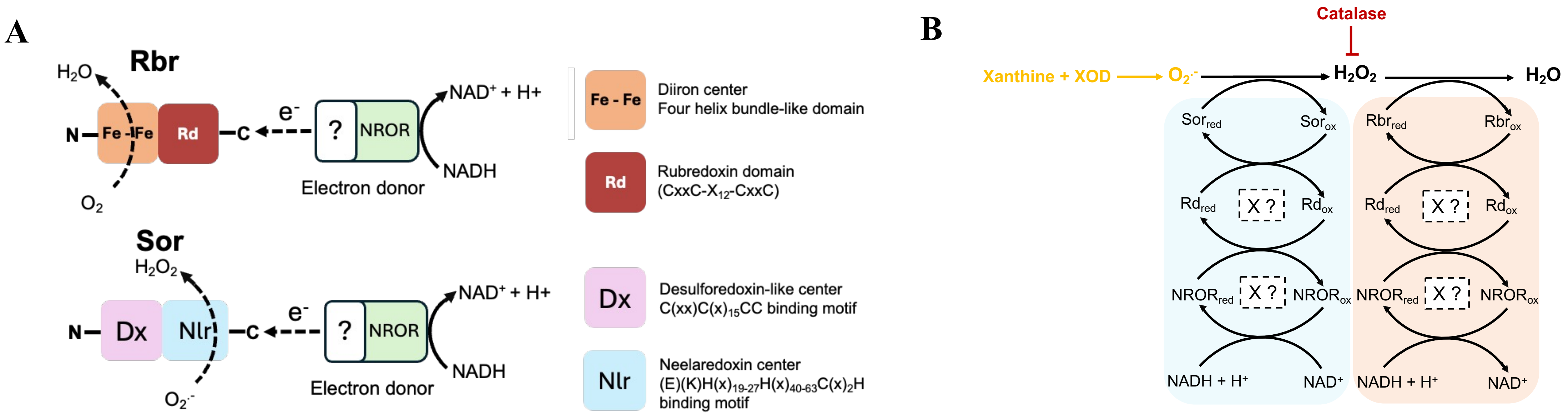

Figure S1. Rbr and Sor representation with associated potential partners

(A) Representation of Rbr and Sor with protein domains involved in O_2-reductase and $O_2^{\cdot-}$-reductase activity, respectively, and potential partners. (B) Oxidation pathway representation with Rbr and Sor with substrates used for biochemical experiments. Rd : Rubredoxin, red : reduced, ox : oxidized, NROR : NADH-Rd oxidoreductase.

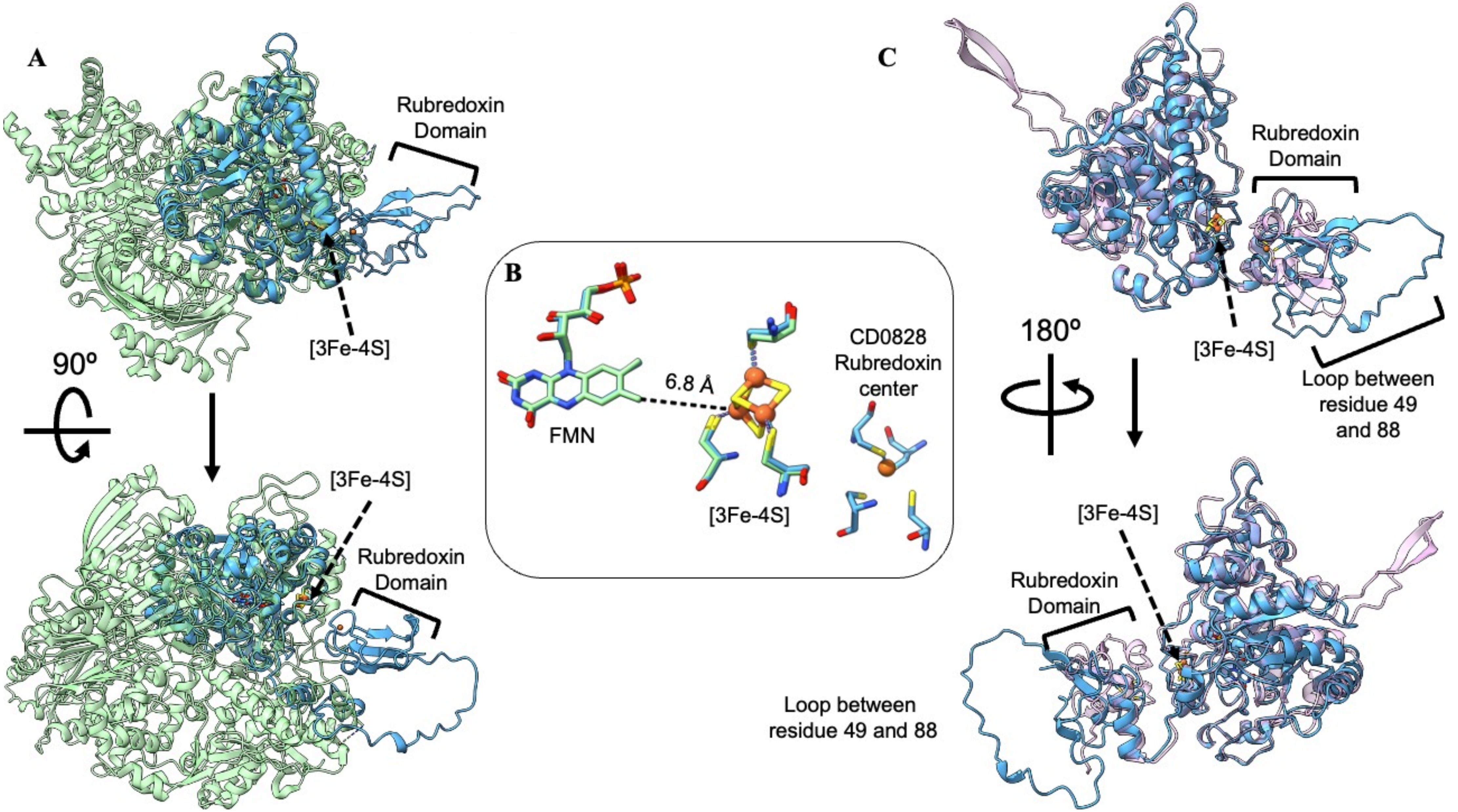

<u>Figure S2: CD0828 structure prediction using AlphaFold3.</u> (A) Structure superposition of the CD0828 model (blue) with the crystallographic structure of glutamate synthase from *Synechocystis sp. PCC 6803* (PDB 1OFD, green) is shown in two different spatial orientations, highlighting the different sizes of the two proteins. (B) Highlight of the relative position of the FMN and $[3Fe\text{-}4S]^{1+/0}$ clusters of both structures as well as the Rd center of CD0828. (C) Structure superposition of the same CD0828 model as in (A) (blue) with the AlphaFold model of the putative glutamate synthase from *Methanocaldococcus jannaschii* (Uniprot Q58746, pink) in two different spatial orientations. CD0828 model structure was prepared with AlphaFold 3 and the cofactor prediction was performed with AlphaFill. Figures were prepared and displayed using ChimeraX.

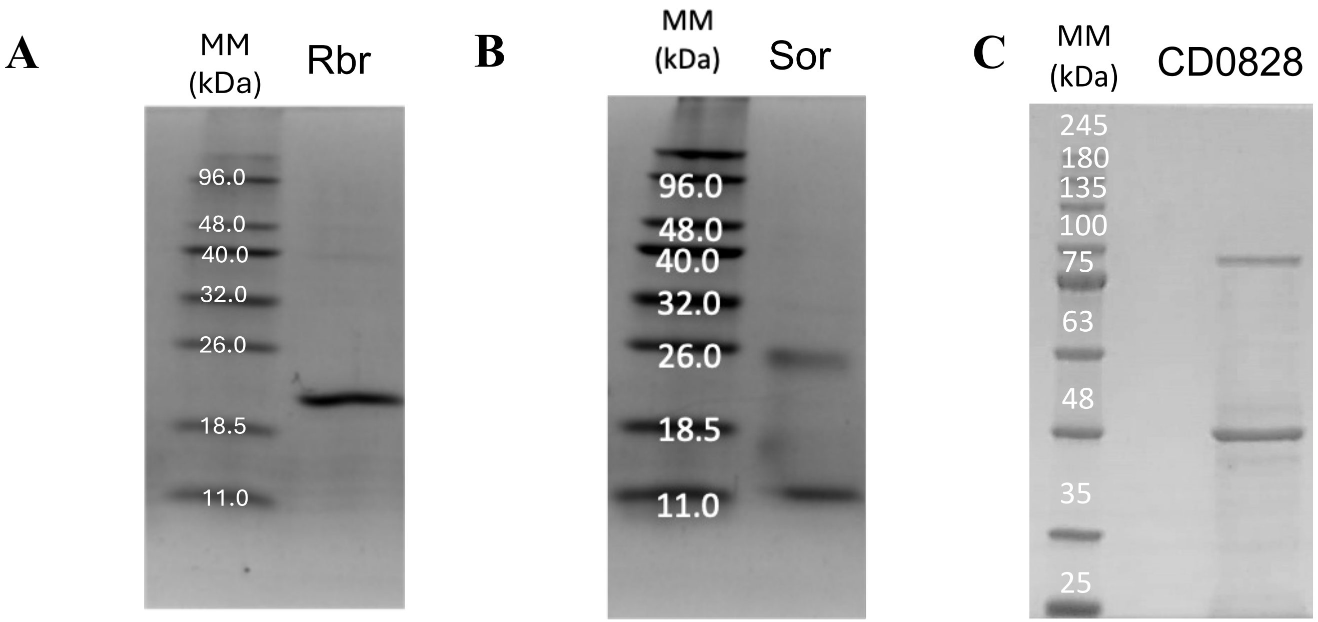

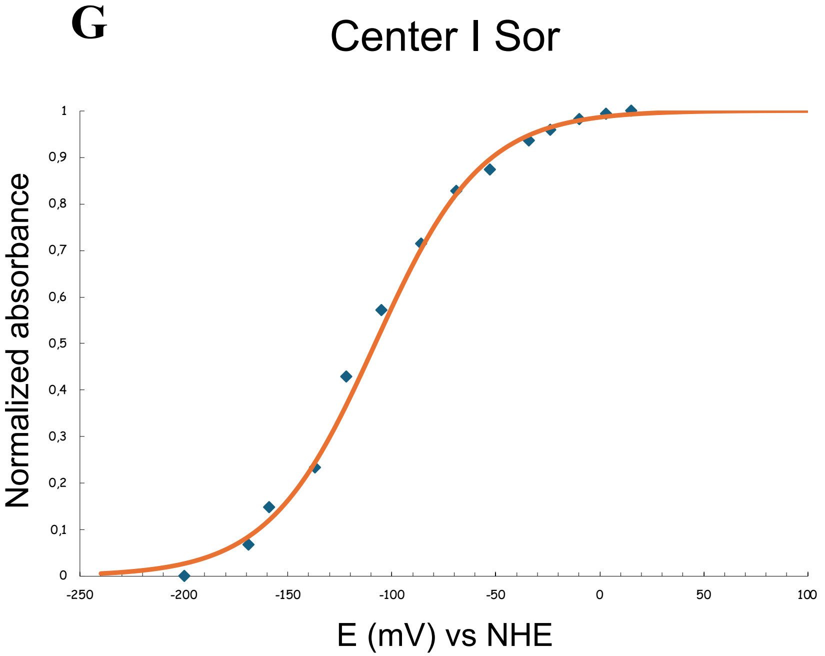

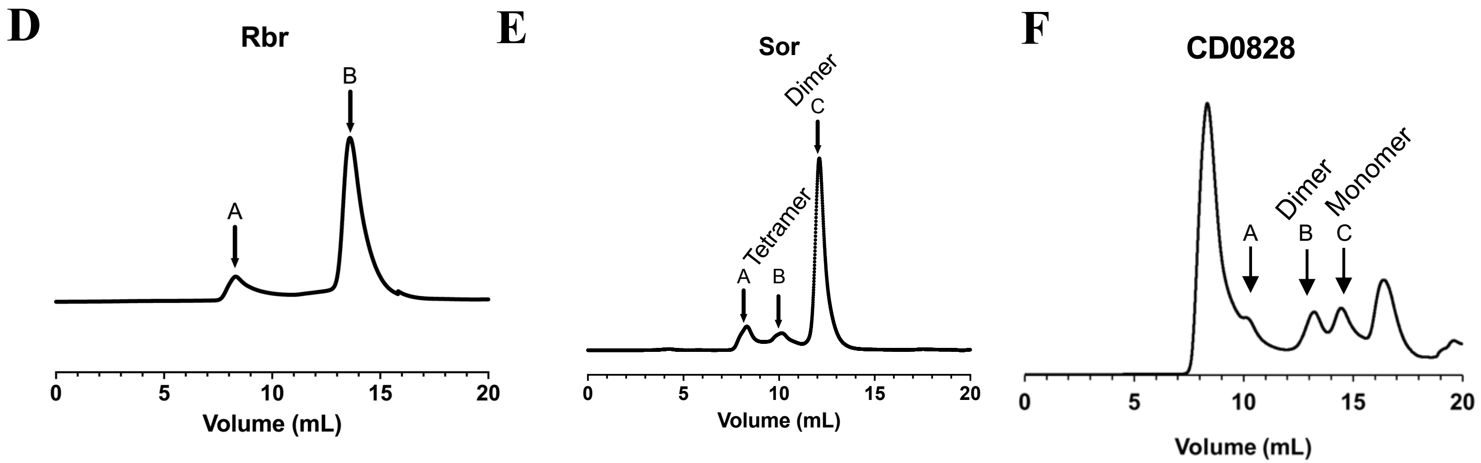

<u>Figure S3: Purification and analysis of Rbr, Sor and CD0828.</u>

(A) SDS-PAGE gel of purified Rbr protein. (B) SDS-PAGE gel of purified Sor protein. (C) SDS-PAGE gel of purified CD0828 protein. Low-range molecular mass markers are indicated (A-C). (D-F) Size exclusion chromatogram of Rbr, Sor or CD0828 monitored at 280 nm. We used a Superdex S200 10/300 GL column previously equilibrated with buffer A containing 150 mM NaCl. (D) Rbr profile. Arrow A, dextran blue elution band; arrow B, protein's elution bands. (E) Sor profile. Arrow A, dextran blue elution band; arrow B protein's elution bands as a tetramer and arrow C as a dimer. (F) CD0828 profile. Arrow A, dextran blue elution band; arrow B protein's elution bands as a dimer and arrow C as a monomer. (G) Anaerobic redox titration curve of center I of Sor. Normalized intensities measured at 490 nm to follow the center I (Dx-like) of Sor. Protein concentration was 30 µM and the experiment was performed in 50 mM Tris-HCl (pH 7.5) containing 18% glycerol. The solid lines correspond to fit to the experimental data using the Nernst equations adjusted as described in Materials and Methods, with the following a reduction potential of -120 ± 5 mV.

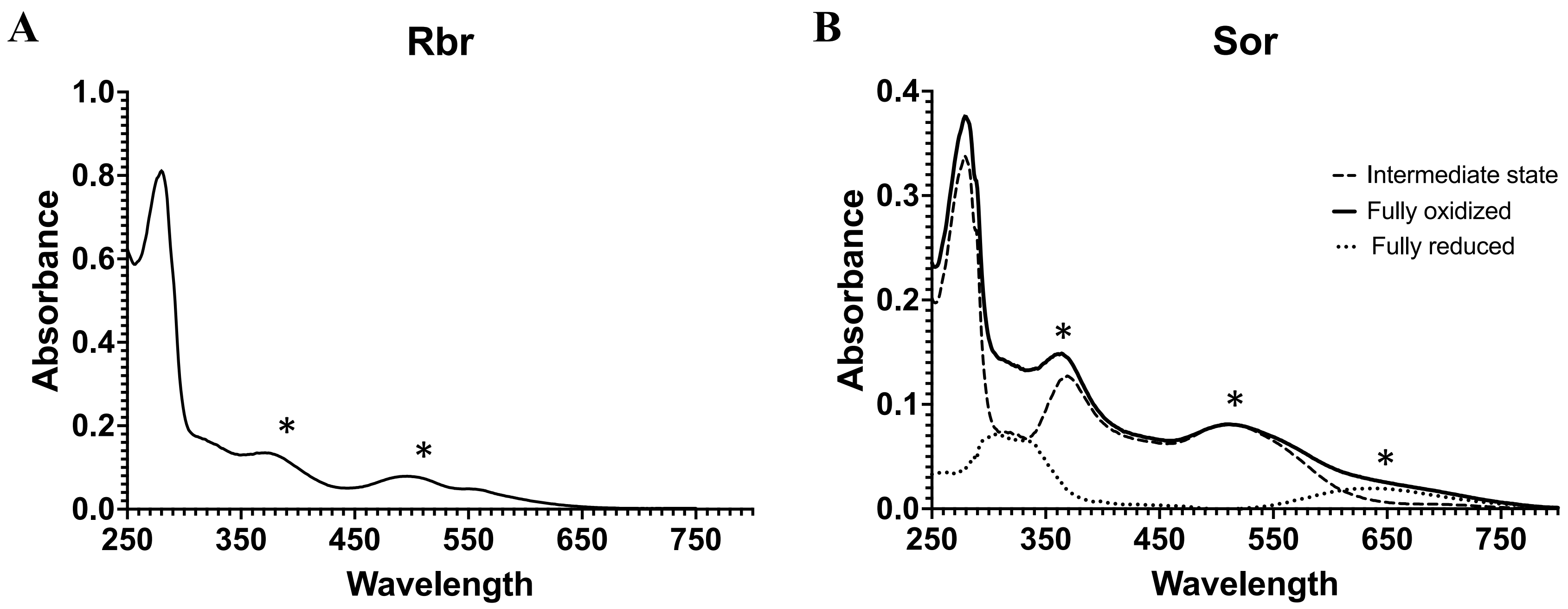

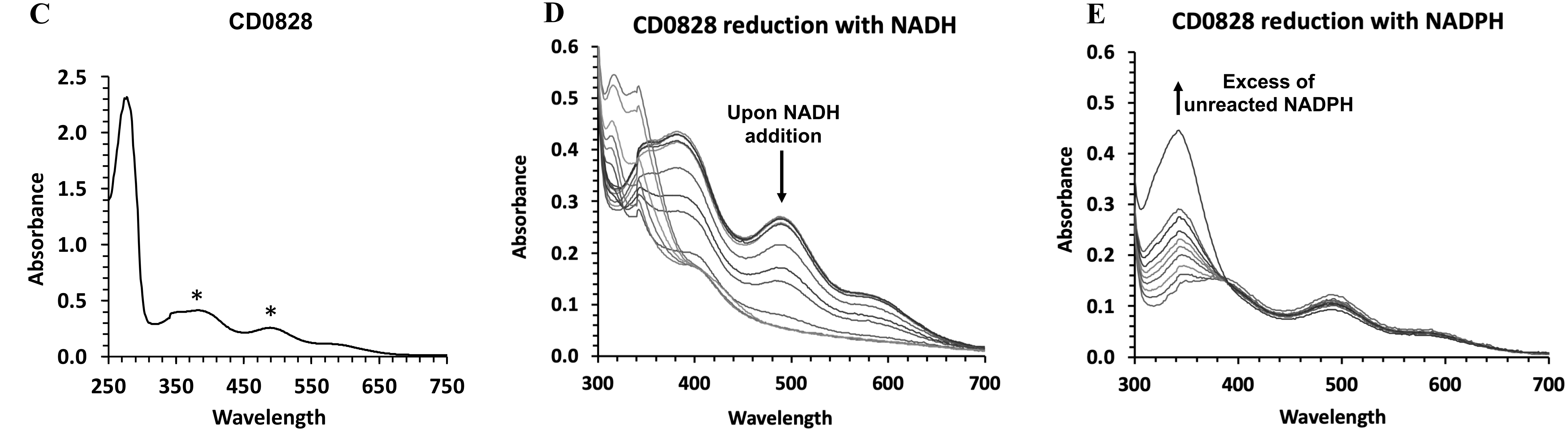

Figure S4: UV-visible spectra of Rbr, Sor and CD0828.

The full lines represent the spectra of Rbr (A), Sor (B) and CD0828 (C) in the fully oxidized form. For Sor and Rbr spectra, the protein concentration was 30 µM in 50 mM Tris-HCl (pH 7.5) containing 18% glycerol whereas for CD0828 it was 40 µM of the protein in 100 mM MOPS pH 7.5 and 150 mM NaCl. (B) The dashed line represents the spectrum of the semi-reduced, "pink" form of Sor (center I oxidized and center II reduced), whereas the dotted line represents the spectral contribution of the center II to the oxidized state spectrum and was obtained after subtracting the spectrum of the semi-reduced form to the fully oxidized one. (D-E) anaerobic reduction of CD0828 with stepwise additions of sub-stoichiometric amounts of NADH (D) or NADPH (E) without the addition of FMN. Reagents' concentration in the assay were 20 µM of CD0828 and 200µM NAD(P)H in 100 mM MOPS, pH 7.5 and 150 mM NaCl.

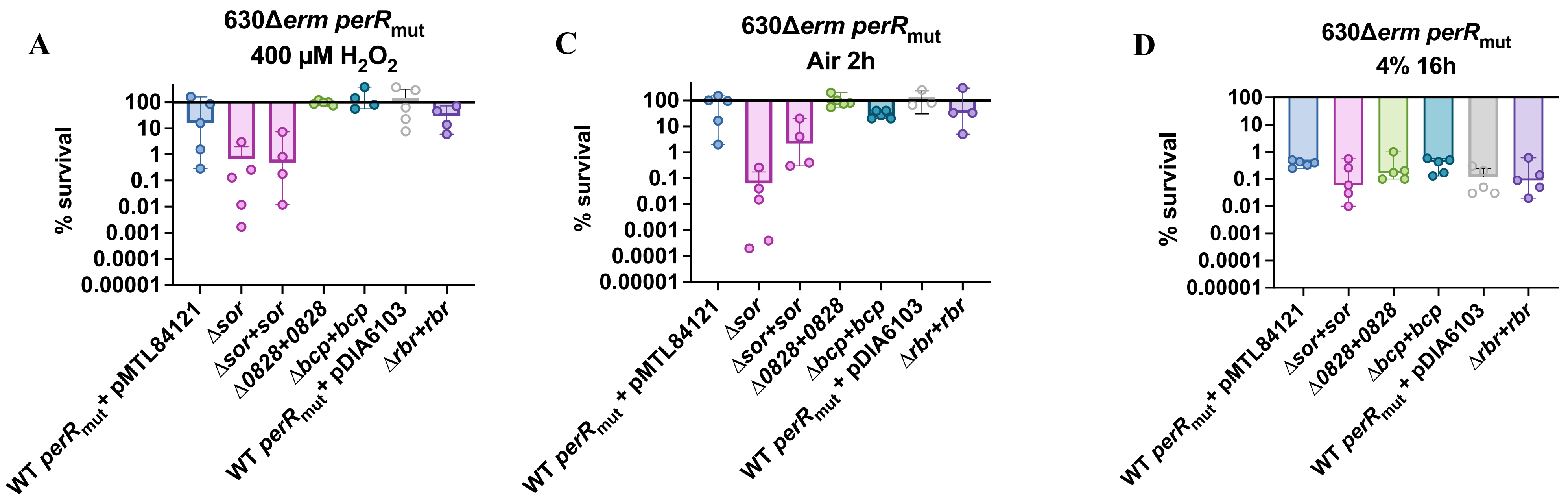

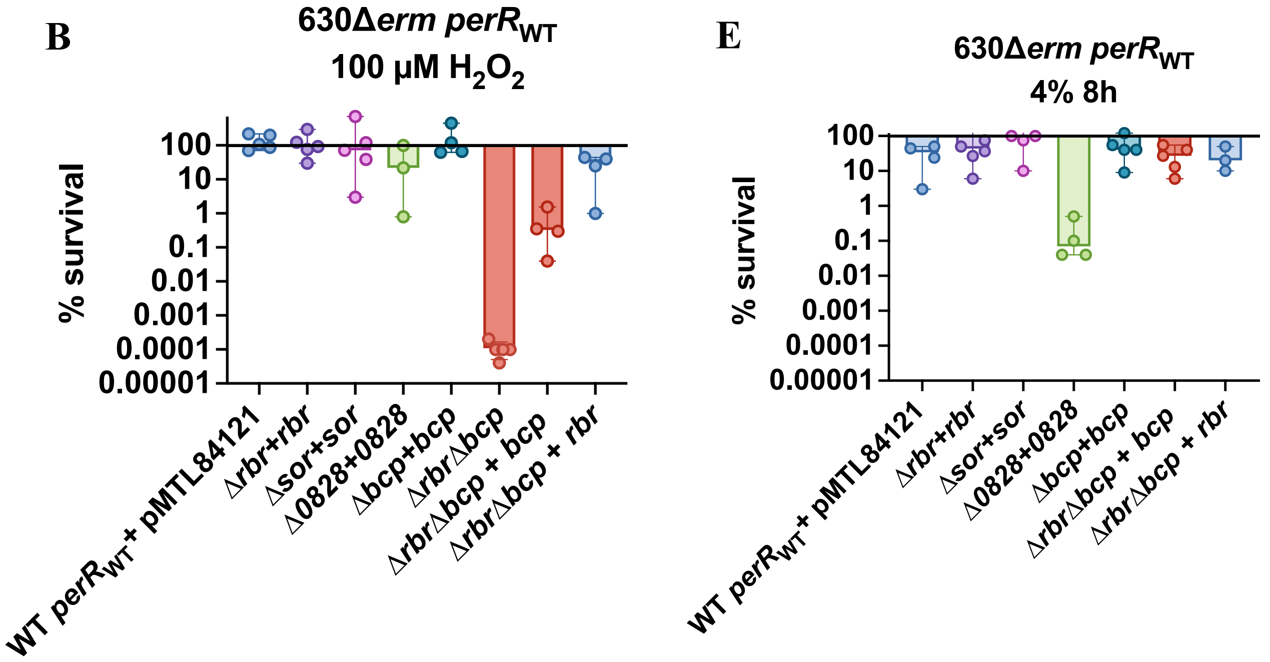

Figure S5: Survival of complemented strains under stress exposure.

(A-B) WT, single mutants inactivated for *rbr*, *sor*, *CD0828* or *bcp* in $perR_{\mathrm{mut}}$ (A) and $perR_{\mathrm{WT}}$ (B) backgrounds of the 630Δ*erm* strain were exposed for 30 minutes to 400 µM and 100 µM of H_2O_2, respectively in a glycylglycine buffer. Serial dilutions were spread in BHI plates that were incubated in anaerobiosis for 24 h. CFUs were determined before and after H_2O_2 exposure and the ratio was calculated to obtain the percentage of survival. (C) Serial dilutions of the WT and single mutants inactivated for *rbr*, *sor*, *CD0828* or *bcp* in $perR_{\mathrm{mut}}$ background were spotted on TY Tau plates. Plates were incubated either in anaerobiosis for 24 h, or in presence of air during 2 h followed by an incubation of 24 h in anaerobiosis. CFUs were determined for each experiment and the percentage of survival after air exposure relative to the survival in anaerobiosis was plotted. (D-E) Serial dilutions of the WT and single mutants inactivated for *rbr*, *sor*, *CD0828* or *bcp* in $perR_{\mathrm{mut}}$ (D) and $perR_{\mathrm{WT}}$ (E) backgrounds were spotted on TY Tau plates. Plates were incubated in presence of 4% O_2 for 16 h (630Δ*erm* $perR_{\mathrm{mut}}$) or 8 h (630Δ*erm* $perR_{\mathrm{WT}}$) followed by 24 h in anaerobiosis before CFUs counting. At least 3 biological replicates were performed per experiment. For all plots, mean with standard deviation are shown. Ordinary one-way ANOVA was performed followed by Dunett's multiple comparison test with the 630Δ*erm* strain $perR_{\mathrm{mut}}$ or $perR_{\mathrm{WT}}$. *: p-value <0.05; **: p-value<0.01. For all assays, ordinary one-way ANOVA was performed followed by Dunett's multiple comparison test with the 630Δ*erm* strain $perR_{\mathrm{mut}}$ or $perR_{\mathrm{WT}}$.

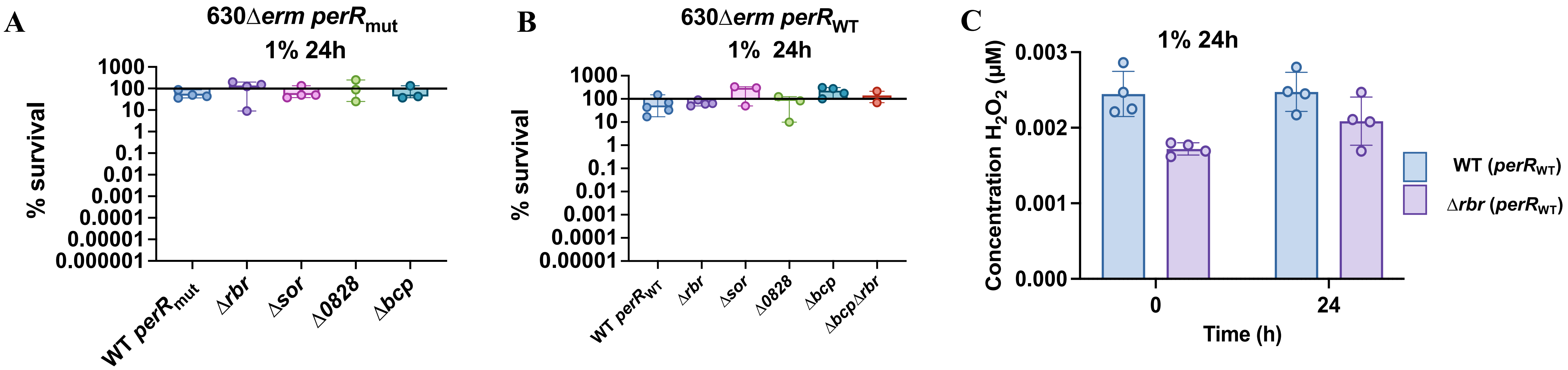

Figure S6. Survival of strains during growth in the presence of 1% O_2.

(A-B) Serial dilutions of the WT and single mutants inactivated for *rbr1*, *sor*, *CD0828* or *bcp* in *perR*$_{mut}$ (A) and *perR*$_{WT}$ (B) backgrounds and the double Δ*rbr1* Δ*bcp* mutant of the 630Δ*erm perR*$_{WT}$ strain (B) were spotted on TY Tau plates. Plates were incubated either in anaerobiosis for 24 h, or in presence of 1% O_2 during 24 h followed by an incubation of 24 h in anaerobiosis. CFUs were determined for each experiment and the percentage of survival after air exposure relative to the survival in anaerobiosis was plotted. For all assays, ordinary one-way ANOVA was performed followed by Dunett's multiple comparison test with the 630Δ*erm* strain *perR*$_{mut}$ or *perR*$_{WT}$. (C) Measurements of H_2O_2 concentration (µM) in supernatants of the *perR*$_{WT}$ and associated single mutant Δ*rbr* in glycylglycine buffer after 24 h at 1% O_2. The parental strains are indicated in light blue and the isogenic single Δ*rbr* mutant in purple.

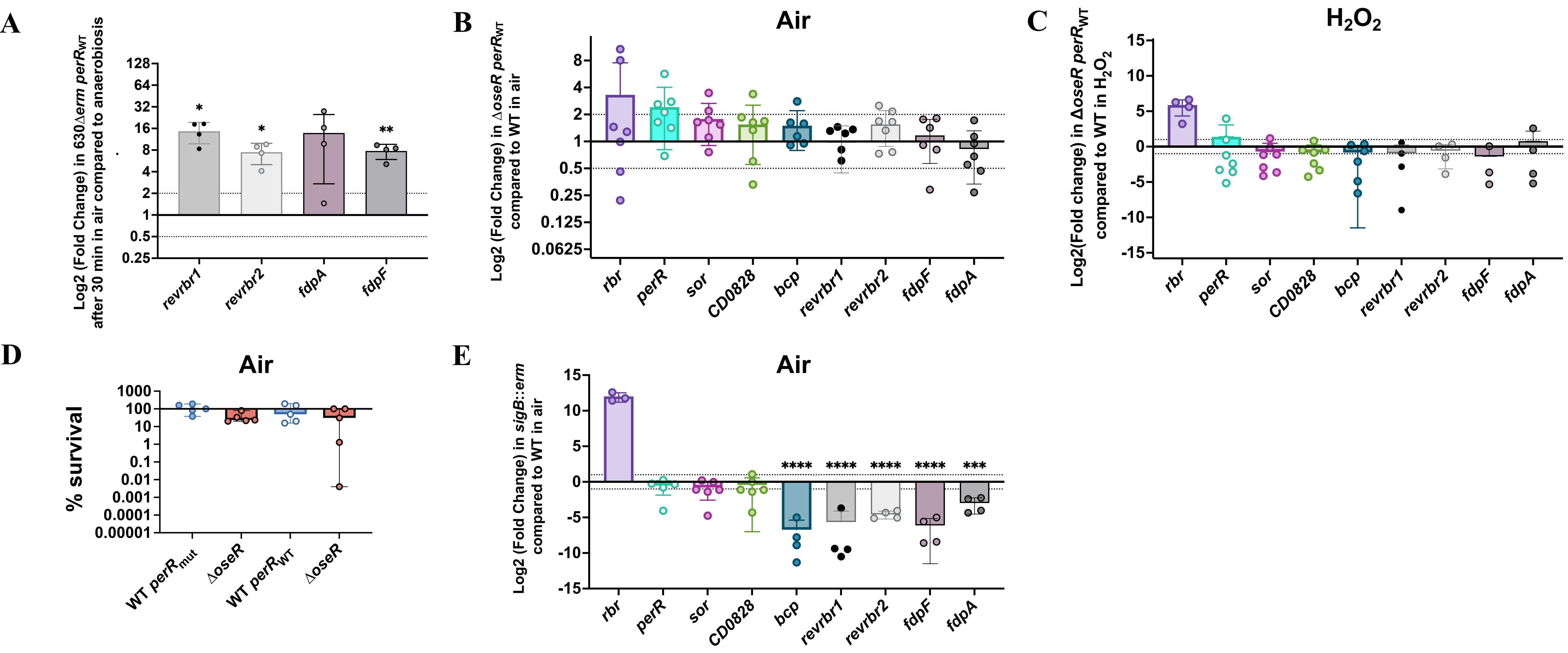

Figure S7. Induction and regulation of genes encoding ROS detoxification systems under air exposure.

Differential expression of the *rbr*, *perR*, *sor*, *CD0828*, *bcp, revrbr1*, *revrbr2*, *fdpF* and/or *fdpA* genes was evaluated by RT-qPCR. We compared expression of the *revrbr1*, *revrbr2*, *fdpF* and *fdpA* genes after 30 min in air compared to anaerobiosis (A). We also compared the expression of all the genes in the Δ*oseR* $perR_{WT}$ mutant and the parental strain after 30 min in air (B) or H_2O_2 (C) in log(2)FC, the $perR_{WT}$ *sigB::erm* $perR_{WT}$ mutant in log2(FC) and the parental strain after 30 min in air (E). *pgi* or *gyrA* were used as reference genes. Experiments were performed at least in 4 biological replicates. Mean and standard deviation (SD) are shown. One sample t-tests were used with comparison of the fold change to 1. ns: not significant, *: p-value <0.05 ; **: p-value<0.01. Finally, serial dilution of the WT and Δ*oseR* mutant in the $perR_{WT}$ and $perR_{mut}$ backgrounds were spotted on TY Tau plates and incubated 1 h ($perR_{WT}$) or 2 h ($perR_{mut}$) in air followed by 24 h in anaerobiosis before CFUs counting (D).
