# Supplementary material for: Defense arsenal of the strict anaerobe Clostridioides difficile against reactive oxygen species encountered during its infection cycle
Source: mBio. 2025 Mar 20;16(4):e03753-24. doi: 10.1128/mbio.03753-24 (PMC11980386; doi:10.1128/mbio.03753-24)
Supplement: Supplemental tables — Tables S1 and S2. [file mbio.03753-24-s0002.docx]

**Table S1 Mutants and plasmids**

| **Strains** | **Genotype** | **Origin** |
| --- | --- | --- |
| ***E. coli*** |  |  |
| HB101 (RP4) | *supE*44 *aa*14 *galK2 lacY1* ∆(*gpt-proA*) 62 *rpsL20* (Str^R^)*xyl-5 mtl-1 recA13* ∆(*mcrC-mrr*) *hsdS*_B_(r_B_^-^m_B_^-^) RP4 (Tra^+^ IncP Ap^R^ Km^R^ Tc^R^) | Laboratory stock |
| NEB10 | Δ(*ara-leu*) *7697 araD139 fhuA* Δ*lacX74 galK16 galE15 e14-* ϕ*80dlacZ*Δ*M15* *recA1 relA1 endA1 nupG rpsL* (Str^R^) *rph spoT1* Δ(*mrr-hsdRMS-mcrBC*) | NewEnglands BioLabs |
| BLI5 | BL21 DE3 (F*^-^ ompT gal dcm lon hsdS_B_*(*r_B_^-^ m_B_^-^*) λ(DE3 [*lacI lacUV5-T7p07 ind1 sam7 nin5*]) [*malB^+^*]_K-12_(λ^S^)) + pDIA17 (*lacI^q^*) | Laboratory stock |
| EC1469 | BLI5 (pET22-*rbr*) | This work |
| EC1470 | BLI5 (pET22-*sor*) | This work |
| EC1631 | BLI5 (pET22-*CD0828*_opt_) | This work |
| ***C. difficile*** |  |  |
| 630∆*erm* | *perR*_mut_ (*perR*_T41A_) | Laboratory stock |
| CDIP1887 | 630∆*erm* *perR*_mut_ ∆*rbr* | This work |
| CDIP1839 | 630∆*erm* *perR*_mut_ ∆*sor* | This work |
| CDIP2093 | 630∆*erm* *perR*_mut_ ∆*CD0828* | This work |
| CDIP2016 | 630∆*erm* *perR*_mut_ ∆*bcp* | This work |
| CDIP1412 | 630∆*erm* *perR*_mut_ ∆*oseR* | Caulat et al., 2024 |
| CDIP546 | 630∆*erm* *perR*_mut_ *sigB::erm* | Kint et al., 2017 |
| CDIP1942 | 630∆*erm* *perR*_WT_ | Laboratory stock |
| CDIP2041 | 630∆*erm perR*_WT_ ∆*rbr* | This work |
| CDIP1973 | 630∆*erm* *perR*_WT_ ∆*sor* | This work |
| CDIP2092 | 630∆*erm* *perR*_WT_ ∆*CD0828* | This work |
| CDIP2069 | 630∆*erm* *perR*_WT_ ∆*oseR* | This work |
| CDIP2332 | 630∆*erm* *perR*_WT_ ∆*bcp* | This work |
| CDIP2327 | 630∆*erm* *perR*_WT_ ∆*rbr* ∆*bcp* | This work |
| CDIP1945 | 630∆*erm* *perR*_WT_ ∆*fdpF* | This work |
| CDIP1949 | 630∆*erm* *perR*_WT_ *sigB::erm* | Laboratory stock |
| CDIP2127 | 630∆*erm* *perR*_WT_ ∆rev*rbr2* | This work |
| CDIP2131 | 630∆*erm* *perR*_WT_ ∆rev*rbr1* | This work |
| CDIP871 | 630∆*erm* *perR*_mut_ pMTL84121 | Laboratory stock |
| CDIP2318 | 630∆*erm* *perR*_mut_ pDIA6103-P_tet_-*rbr* | This work |
| CDIP1880 | 630∆*erm* *perR*_mut_ ∆*sor* pMTL84121-P*_sor_-sor* | This work |
| CDIP2105 | 630∆*erm* *perR*_mut_ ∆*CD0828* pMTL84121-P*_rbr_-CD0828* | This work |
| CDIP2241 | 630∆*erm* *perR*_mut_ ∆*bcp* pMTL84121-P*_bcp_-bcp* | This work |
| CDIP2135 | 630∆*erm* *perR*_WT_ pMTL84121 | This work |
| CDIP2099 | 630∆*erm* *perR*_WT_ ∆*rbr* pMTL84121-P*_rbr_-rbr* | This work |
| CDIP2100 | 630∆*erm* *perR*_WT_ ∆*sor* pMTL84121-P*_rbrr_-sor* | This work |
| CDIP2105 | 630∆*erm* *perR*_WT_ ∆*CD0828* pMTL84121-P*_rbr_-CD0828* | This work |
| CDIP2335 | 630∆*erm* *perR*_WT_ ∆*bcp* pDIA84121-P*_bcp_-bcp* | This work |
| CDIP2288 | 630∆*erm* *perR*_WT_ pFT47-P*_oseR_*-SNAP | This work |
| CDIP2301 | 630∆*erm* *perR*_WT_ *sigB*::*erm* pFT47-P*_oseR_*-SNAP | This work |
| **Plasmids** | **Characteristics** | **Origins** |
| pMTL84121 | Replicative plasmid in both *E. coli* and *C. difficile* which can be transfered by conjugation | Heap *et al*. 2009 |
| pDIA6103 | Replicative plasmid in both *E. coli* and *C. difficile* with a P_tet_ promoter | Soutourina et al., 2013 |
| pMSR-ACE | Plasmid for gene inactivation by ACE in *C. difficile* | Laboratory stock |
| pFT47 | SNAP, Cm^R^-Tm^R^ | Pereira et al., 2013 |
| pET22 | Overexpression of protein in *E. coli,* Amp^R^ | Novagen |
| pDIA7121 | pMTL84121-P*_rbr_*-*rbr* | This work |
| pDIA7125 | pMTL84121-P*_rbr_*-*sor* | This work |
| pDIA7257 | pMTL84121-P*_rbr_*-*CD0828* | This work |
| pDIA7291 | pMTL84121-P*_bcp-_bcp* | This work |
| pDIA7327 | pDIA6103-P_tet_-*rbr* | This work |
| pDIA6887 | pMSR ACE *revrbr1* | Kint et al, 2020 |
| pDIA6888 | pMSR ACE *revrbr2* | Kint et al, 2020 |
| pDIA6893 | pMSR ACE *fdpF* | Kint et al, 2020 |
| pDIA7120 | pMSR ACE *rbr* | This work |
| pDIA7123 | pMSR ACE *sor* *perR*_mut_ | This work |
| pDIA7139 | pMSR ACE *sor* *perR*_WT_ | This work |
| pDIA7248 | pMSR ACE *CD0828* | This work |
| pDIA7184 | pMSR ACE *bcp* | This work |
| pDIA6555 | pDIA6103 miniarray-*hfq* | Maikhova et al, 2020 |
| pDIA7147 | pDIA6103 miniarray-*rbr* ACE ∆*rbr* | This work |
| pDIA7315 | pFT47-P*_oseR_*-SNAP | This work |
| pDIA7151 | pET22-*rbr* | This work |
| pDIA7153 | pET22-*sor* | This work |
| pDIA7276 | pET22-*CD0828*-opt | This work |

the *rbr* gene corresponds to *CD0825*

**Table S2 Primers**

| **Name** | **Sequence** | **Function** |
| --- | --- | --- |
| **Mutant construction** | |  |
| LC2 | GTTTATTTTGGGGTTTTAGATTAACTATATGGAATGTAAATTCAACAGAGGAAAATTTAAAGGATGCAGCAGCAGGTGGTTTTAGATTAACTATATGGAATGTAAATCTATAAGTTTTAA | Insertion protospacer *rbr* |
| LC3 | TTAAAACTTATAGATTTACATTCCATATAGTTAATCTAAAACCACCTGCTGCTGCATCCTTTAAATTTTCCTCTGTTGAATTTACATTCCATATAGTTAATCTAAAACCCCAAAATAAAC | Insertion protospacer *rbr* |
| LC4 | TGGTCATGAGATTATCAAAAGGATTTGGCATAAAAAATGGAGCA | ACE *bcp* FI |
| LC5 | TCTCTTGTGCATTTGTGTCTGGTGGTGCTTTTGTTCCTATACTCAA | ACE *bcp* FI |
| LC6 | CCAGACACAAATGCACAAGAGA | ACE *bcp* FII |
| LC7 | ATCGTAGAAATACGGTGTTTTTTGTCATCATACCTCTAGCTTTC | ACE *bcp* FII |
| IMV1307 | TGGTCATGAGATTATCAAAAGGGTGTAGGGATTGCATTTG | ACE *rbr* FI |
| IMV1308 | TCCTGCCATTAAATTCTTTTC | ACE *rbr* FI |
| IMV1309 | GAAAAGAATTTAATGGCAGGACCCAGTATGTGACCATC | ACE *rbr* FII |
| IMV1310 | ATCGTAGAAATACGGTGTTTTTTCACTACACATAAACTCACC | ACE *rbr* FII |
| IMV1311 | TGGTCATGAGATTATCAAAAGGGAGATGAAGAAGTTGTTTGG | ACE *sor* FI |
| IMV1312 | TTCACTACACATAAACTCACC | ACE *sor* FI |
| IMV1313 | GGTGAGTTTATGTGTAGTGAAGAGTCAAAAAGGAGGCT | ACE *sor* FII |
| IMV1330 | ATCGTAGAAATACGGTGTTTTTTCACTACACATAGCTGTCTTATTTTG | ACE *sor* FII |
| IMV1338 | AAAGGCCTGGGTGTAGGGATTGCATTTG | 5’ ACE *rbr* StuI |
| IMV1339 | AAAGGCCTCACTACACATAAACTCACC | 3’ ACE *rbr* StuI |
| IMV1519 | TGGTCATGAGATTATCAAAAGGTTTCTAAACAACGAGAACTGATTTT | ACE *CD0828* FI |
| IMV1520 | AATTGACATAATTATTTTCTCCTTATAT | ACE *CD0828* FI |
| IMV1526 | ATATAAGGAGAAAATAATTATGTCAATTGGTCATAAAGATTTAAGTGTTGATG | ACE *CD0828* FII |
| IMV1522 | ATCGTAGAAATACGGTGTTTTTTACAAATTCCACTATGAGAACATC | ACE *CD0828* FII |
| CA36 | GGAATGTAAATCTATAAGTTTTAATAAAACT | Inv PCR pDIA6555 |
| CA37 | TAATCTAAAACCCCAAAATAAACTTAGTAT | Inv PCR pDIA6555 |
| **Complementation** | | |
| LC10 | GAGCAAGGCAAGACCGATCTATCATTTAACCTTGTTTTAAG | 5’ *bcp* |
| LC11 | CGATTAAGTTGGGTAACGCCAG AAGTTAAGATTGCAGGGTTTC | 3’ *bcp* |
| LC12 | CGTTAACAGATCTGAGCTCCTAAAAAGGGAGGAATTAATTATGA | 5’*rbr-*pMTL84121 |
| LC13 | TATAGGATCCTCTCGAGAAGGCCTTAATTTTTAATAATTTTCAGCTTTTATAT | 3’*rbr-*pMTL84121 |
| IMV1320 | AATTAATTCCTCCCTTTTTAATAATAG | PCR P*_rbr_* |
| IMV1318 | GAGCAAGGCAAGACCGATCTTGCAATAGGTATAGCGACAA | PCR P*_rbr_* |
| IMV1321 | CTATTATTAAAAAGGGAGGAATTAATTCCAGTATGTGACCATCCAAAA | 5’ *perR* |
| IMV1322 | CGATTAAGTTGGGTAACGCCAGTTCACTACACATAAACTCACC | 3’ *perR* |
| IMV1334 | CTATTATTAAAAAGGGAGGAATTAATTGTAAAAGGTGTAGCCAAGTAGG | 5’ *sor* |
| IMV1335 | CGATTAAGTTGGGTAACGCCAGTAGCCTCCTTTTTGACTCAATTTATTT | 3’ *sor* |
| IMV1523 | CTATTATTAAAAAGGGAGGAATTAATTAGTCAAAAAGGAGGCTATAAATG | 5’ *CD0828* |
| IMV1524 | CGATTAAGTTGGGTAACGCCAGGCTTCATACAAAAAAGAGTGAC | 3’ *CD0828* |
| IMV1653 | AAGGCCTATTATTAAAAAGGGAGGAATTAATTAT | 5’ *rbr*-StuI pDIA6103 |
| IMV1654 | GGGGGATCCATTCCTTTTTAATAATGATAATTTTTAAT | 3’ *rbr*-BamHI pDIA6103 |
| IMV993 | CTGGCGTTACCCAACTTAATCG | 3’ MCS pMTL84121 |
| CM13 | GATCGGTCTTGCCTTGCTC | 5’ MCS pMTL84121 |
| **qRT PCR** | | |
| IMV782 | AGGGCGTCCAATAGGTGTTA | *CD0828* |
| IMV783 | TCCACCACGACCATCAATAG |  |
| IMV784 | ACTGAACAAGGTGGTCAACG | *sor* |
| IMV785 | CAGTTCAGCCTTCCATAATCC |  |
| QRTBD269 | TGCAGGAGAGTCAGAAGCAA | *rbr* |
| QRTBD270 | GCATGTTCCTTTTCATTGTTAGC |  |
| LC16 | GATGTACACGTCAAGCTTGTG | *bcp* |
| LC17 | CAACTAAGTCAGGGTCAGAAAG |  |
| IMV693 | TGGTTCAAAACCCTCCTAGTAAA | *oseR* |
| IMV694 | GACCCAATTCTCTATATTTCATACCA |  |
| IMV575 | CCAAGGCAAGATAGCAGGAG | *fdpA* |
| IMV576 | CTGCACCAAATGCCATACAC |  |
| IMV668 | TGCCCTGTATGTGGAGCTAA | *fdpF* |
| IMV669 | CCAGCTGCTCCATTTCCTAC |  |
| IMV670 | ACATGAAGGAGATGCTGCAC | *revrbr1*/*revrbr2* |
| IMV671 | GTGCTCATCAGCCCAATTTT | *revrbr1* |
| IMV672 | ATGTTCATCAGCCCAAACCA | *revrbr2* |
| QRTBD3 | TTTTGTTGTGTCTATGAACCTTTGT | *gyrA* |
| QRTBD4 | TCCTTTACCAGCTCTTATTTGACTT |  |
| QRTBD43 | CAGATGATGTAGGTGGTCGTTTTT | *pgi* |
| QRTBD44 | AGCAGCAATAGGAAGTAACCCAAC |  |
| **Transcriptional SNAP fusion** | | |
| IMV1103 | CCGAGCTCGAATTCGTAATCATGGT | Amplification of pFT47 for cloning through Gibson  Assembly |
| IMV1563 | CTCGAGGGAGGAACTACTATG |  |
| AL12 | ACCATGATTACGAATTCGAGCTCGGGCAAGTAAAATCAAATATACTATGCCC | pFT47-IMV1103 comp-5’-P*oseR* |
| AL13 | CATAGTAGTTCCTCCCTCGAGATTGTTATACCCATAAATTGTATACTATAACCAA | pFT47-IMV1563 comp-3’-P*oseR* |
| **Overexpression in *E. coli*** | | |
| IMV1369 | AACTTTAAGAAGGAGATATACAT**ATG**AACTTAAAAGGAACTAAAACAGAAA | 5’ *rbr* pET22 |
| IMV1371 | GTGGTGGTGGTGCTCGAGTTTAGAAAATTTCATAGTTAACATTCC | 3’ *rbr* pET22 |
| IMV1372 | AACTTTAAGAAGGAGATATACAT**ATG**TGTAGTGAACAAAAATTTTTTATATGT | 5’ *sor* pET22 |
| IMV1374 | GTGGTGGTGGTGCTCGAGCCTCCTTTTTGACTCAATTTATT | 3’ *sor* pET22 |
| AL01 | AACTTTAAGAAGGAGATATACATATGTCGATCTATAAATGCTCTGTATGTG | 5’ *CD0828*_opt_ pET22 |
| AL02 | GTGGTGGTGGTGCTCGAGCTATACGTGTTGAATGTTTGTATAGTTAGAA | 3’ *CD0828*_opt_ pET22 |
